# Characterization of a Feline Influenza A(H7N2) Virus

**DOI:** 10.3201/eid2401.171240

**Published:** 2018-01

**Authors:** Masato Hatta, Gongxun Zhong, Yuwei Gao, Noriko Nakajima, Shufang Fan, Shiho Chiba, Kathleen M. Deering, Mutsumi Ito, Masaki Imai, Maki Kiso, Sumiho Nakatsu, Tiago J. Lopes, Andrew J. Thompson, Ryan McBride, David L. Suarez, Catherine A. Macken, Shigeo Sugita, Gabriele Neumann, Hideki Hasegawa, James C. Paulson, Kathy L. Toohey-Kurth, Yoshihiro Kawaoka

**Affiliations:** University of Wisconsin–Madison, Madison, Wisconsin, USA (M. Hatta, G. Zhong, Y. Gao, S. Fan, S. Chiba, K.M. Deering, T.J. Lopes, G. Neumann, K.L. Toohey-Kurth, Y. Kawaoka);; National Institute of Infectious Diseases, Tokyo, Japan (N. Nakajima, H. Hasegawa);; University of Tokyo, Tokyo (M. Ito, M. Imai, M. Kiso, S. Nakatsu, Y. Kawaoka);; The Scripps Research Institute, La Jolla, California, USA (A.J. Thompson, R. McBride, J.C. Paulson);; US Department of Agriculture, Athens, Georgia, USA (D.L. Suarez);; The University of Auckland, Auckland, New Zealand (C. A. Macken);; Japan Racing Association, Tochigi, Japan (S. Sugita)

**Keywords:** influenza virus, viruses, influenza, H7N2, feline, zoonotic infection, zoonoses, respiratory infections, New York, United States

## Abstract

During December 2016–February 2017, influenza A viruses of the H7N2 subtype infected ≈500 cats in animal shelters in New York, NY, USA, indicating virus transmission among cats. A veterinarian who treated the animals also became infected with feline influenza A(H7N2) virus and experienced respiratory symptoms. To understand the pathogenicity and transmissibility of these feline H7N2 viruses in mammals, we characterized them in vitro and in vivo. Feline H7N2 subtype viruses replicated in the respiratory organs of mice, ferrets, and cats without causing severe lesions. Direct contact transmission of feline H7N2 subtype viruses was detected in ferrets and cats; in cats, exposed animals were also infected via respiratory droplet transmission. These results suggest that the feline H7N2 subtype viruses could spread among cats and also infect humans. Outbreaks of the feline H7N2 viruses could, therefore, pose a risk to public health.

Influenza A viruses are endemic in humans and enzootic in other mammalian species including swine and horses; occasional infections of other mammalian species including whales, seals, sea lions, felidae in zoos, and other species have been reported (*1*). Reports of influenza A virus infections in dogs and cats were rare until 2004, when equine influenza viruses of the H3N8 subtype caused outbreaks in greyhounds in Florida (*2*). Since then, influenza viruses of the H3N8 and H3N2 subtypes have caused several outbreaks in dogs in the United States and South Korea (*3*–*5*).

Until recently, only 1 major influenza A virus outbreak had been reported in cats (*6*). This changed in December 2016 with the outbreak of low pathogenic avian influenza A viruses of the H7N2 subtype in animal shelters in New York. Approximately 500 cats were infected in December 2016–February 2017; most of which experienced a mild illness with coughing, sneezing, and runny nose from which they recovered fully. Severe pneumonia developed in 1 elderly animal with underlying health issues, which was euthanized. A veterinarian who had treated an infected animal also became infected with the feline influenza A(H7N2) virus and experienced a mild, transient illness, suggesting the potential for these viruses to infect humans. While this manuscript was being prepared, Belser et al. reported that the H7N2 subtype virus isolated from the human case caused a mild disease in mice and ferrets, but was not transmitted among ferrets (*7*). We assessed feline H7N2 subtype viruses isolated from infected cats during the outbreak for their replicative ability, pathogenicity, and transmissibility in mammals; in contrast to the findings recently published by Belser et al. (*7*), we detected productive infection of co-housed ferrets, although with low efficiency. We also conducted extensive pathology and transmission studies in cats, and detected feline virus transmission via respiratory droplets to exposed cats. Our study provides additional data on the risk that the feline H7N2 subtype viruses pose to public health.

## Methods 

### Cells and Viruses

The origins and growth conditions of all cell lines used in this study are described in the Technical Appendix. The feline H7N2 subtype viruses used in this study were isolated from swabs collected from cats with influenza-like symptoms during the outbreak in an animal shelter in New York in December 2016. We obtained A/chicken/New York/22409–4/1999 (H7N2, A/chicken/NY/99) virus from the Agricultural Research Service, US Department of Agriculture (*8*). We deposited the viral gene sequences obtained in this study to GenBank. We amplified the feline virus in Madin-Darby canine kidney (MDCK) cells and the A/chicken/NY/99 virus in 10-day-old embryonated chicken eggs.

### Growth Kinetics of Viruses in Cell Culture

We infected cells with viruses at a 0.005 multiplicity of infection, incubated them for 1 hour at 37°C, washed twice, and cultured with 1× minimal essential medium containing 0.3% bovine serum albumin and trypsin treated with L-1-tosylamide-2-phenylethyl chloromethyl ketone at 33°C and 37°C (37°C and 39°C for chicken embryo fibroblast cells) for various periods. We determined virus titers at the indicated time points by use of plaque assays in MDCK cells. The statistical analyses are described in the Technical Appendix.

### Infection of Animals

To determine the pathogenicity of the viruses in infected mice, we anesthetized three 6-week-old female BALB/c mice (Jackson Laboratory, Bar Harbor, ME, USA) for each virus with isoflurane and inoculated intranasally with 10-fold serially diluted virus in a 50-µL volume. The mice were monitored daily for 14 days and checked for changes in body weight and morbidity and mortality. We euthanized animals if they lost more than 25% of their initial bodyweight. 

To determine the pathogenicity of the viruses in infected ferrets and cats, we inoculated 6-month-old female ferrets (Triple F Farms, Sayre, PA, USA; 3 per group; serologically negative by hemagglutination inhibition assay for currently circulating human influenza viruses), and unvaccinated 4- to 5-month-old female specific-pathogen-free cats (Liberty Research, Waverly, NY, USA; 3 per group) intranasally with 10^6^ PFU of viruses in 0.5 ml of phosphate-buffered saline. We monitored the animals daily for changes in bodyweight, body temperature, and clinical signs for 14 days. 

For virus replication in organs and pathology analyses, we worked with groups of mice (12 per group), ferrets (6 per group), and cats (6 per group).We inoculated the animals intranasally with 10^5^ PFU (mice) in 0.05 ml of phosphate-buffered saline or 10^6^ PFU (ferrets and cats) of viruses in 0.5 ml of phosphate-buffered saline. On days 3 and 6 postinfection, we euthanized 6 mice, 3 ferrets, and 3 cats in each group for pathological analysis and virus titration in organs (by use of plaque assays in MDCK cells). 

### Virus Transmission Studies in Ferrets and Cats

For direct contact transmission experiments, we housed 3 ferrets per group in regular ferret cages and 3 cats per group in large dog transporter cages (Technical Appendix Figure 1), and infected them intranasally with 10^6^ PFU (500 μL) of viruses. One day later, we housed 1 virus-naive animal with each infected animal. We collected nasal washes from the infected ferrets and nasal swabs from the infected cats on day 1 after infection, and from the exposed animals on day 1 after exposure and then every other day (for up to 11 days). We determined virus titers in the nasal washes and swabs by performing plaque assays in MDCK cells. We monitored all animals daily for disease symptoms and changes in bodyweight and temperature for 14 days. 

We performed airborne transmission experiments by using ferret isolators (Showa Science, Tokyo, Japan) (*9*–*11*) or regular cat cages. In these settings, there was no directional airflow from the infected to the exposed animals. We inoculated 3 animals per group intranasally with 10^6^ PFU (500 μL) of viruses. One day after infection, we placed 3 immunologically naive animals (exposed animals) each in a cage adjacent to an infected animal. This setting prevented direct and indirect contact between animals but allowed spread of influenza virus by respiratory droplet. We spaced the ferret cages 5 cm apart and the cat cages 35 cm apart. We monitored the animals and assessed virus titers as described above.

## Results

### Genetic and Phylogenetic Analysis of Feline Influenza(H7N2) Viruses Isolated in Animal Shelters in New York, December 2016

**Figure 1:**
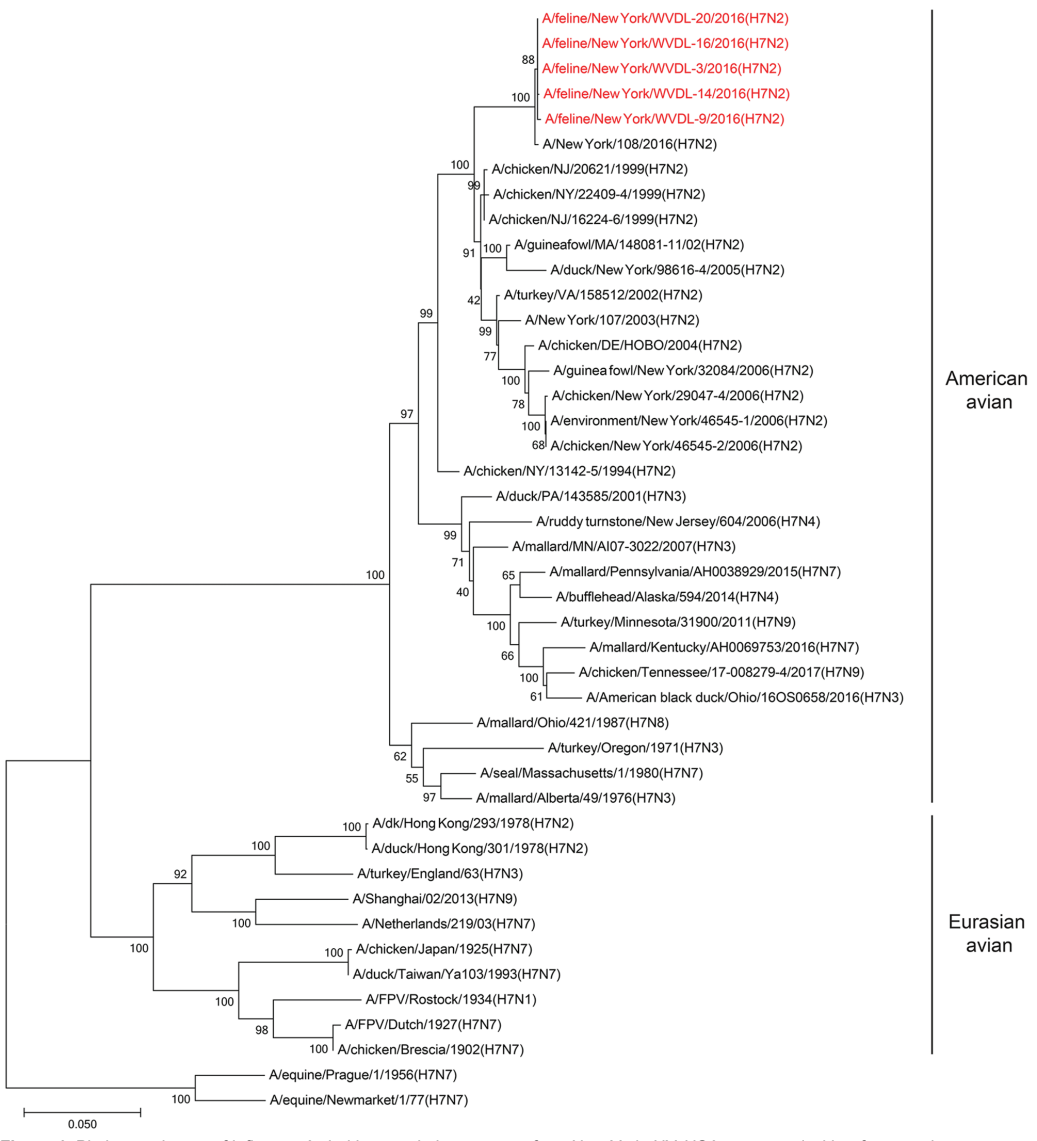
Phylogenetic tree of influenza A viral HA segments. Phylogenetic analysis was performed for selected influenza A viruses representing major lineages. The evolutionary history was inferred using the neighbor-joining method (*12*). The optimal tree with the branch length sum of 1.22521320 is shown. The percentage of replicate trees in which the associated taxa clustered together in the bootstrap test (500 replicates) is shown next to the branches (*13*). The tree is drawn to scale, with branch lengths in the same units as those of the evolutionary distances used to infer the phylogenetic tree. The evolutionary distances were computed using the Tamura 3-parameter method (*14*) and are in the units of the number of base substitutions per site. The analysis involved 44 nt sequences. Codon positions included were 1^st^ + 2^nd^ + 3^rd^ + noncoding. All positions containing gaps and missing data were eliminated. The final dataset contained a total of 1,612 positions. Evolutionary analyses were conducted in MEGA7 (*15*).

We obtained swabs (collected on the same day) from 5 cats that experienced influenza-like symptoms during the outbreak at an animal shelter in New York, NY, in December 2016. After inoculation of these samples into MDCK cells, we isolated 5 pleomorphic influenza A viruses of the H7N2 subtype (Table 1; Technical Appendix Figure 2). The HA consensus sequences of the 5 isolates (established by Sanger sequence analysis) displayed >99.9% similarity at the nucleotide level (Table 1). Phylogenetic analyses demonstrated that the 8 viral RNA segments of the 5 feline H7N2 viruses are most closely related to poultry influenza A(H7N2) viruses detected in the New York area in the late 1990s through early 2000s (Figure 1; Technical Appendix Figures 3–9), suggesting that the 2016 feline H7N2 virus isolates descended from viruses that circulated more than a decade ago in the northeastern United States.

**Table 1:** Amino acid differences among feline H7N2 virus isolates*

Virus	Amino acid positions in the viral proteins†							
	PB2	PB1-F2	PA		NA			NS2
	448	42	57		40	62		74
A/feline/New York/WVDL-3/2016	**S**	**C**	**Q**		**Y**	**C**		D
A/feline/New York/WVDL-9/2016	N	Y	R		H	**C**		**E**
A/feline/New York/WVDL-14/2016	**S**	**C**	**Q**		**Y**	**C**		**E**
A/feline/New York/WVDL-16/2016	**S**	**C**	**Q**		**Y**	F		**E**
A/feline/New York/WVDL-20/2016	**S**	**C**	**Q**		**Y**	**C**		D

*Consensus sequences among the 5 H7N2 subtype viruses are shown in bold.  † Amino acids: C, cysteine; D, aspartic acid; E, glutamic acid; F, phenylalanine; H, histidine; L, leucine; N, asparagine; Q, glutamine; R, arginine; S, serine; Y, tyrosine. Viral proteins: NA, neuraminidase; NS, nonstructural protein; PB, polymerase basic.

The HA protein of the 2016 feline H7N2 subtype virus encodes a single arginine residue at the hemagglutinin cleavage site (PEKPKPR↓G; the arrow indicates the cleavage site that creates the HA1 and HA2 subunits), indicative of low pathogenicity in chickens. Antigenically, A/feline/New York/WVDL-14/2016 (A/feline/NY/16) differs from other, closely related H7 viruses (Technical Appendix Table 1); for example, its HA deviates by 27 aa from the closely related A/chicken/NY/22409–4/1999 HA. The neuraminidase (NA) and ion channel (M2) proteins of the H7N2 viruses do not encode amino acids that confer resistance to neuraminidase or ion channel inhibitors. Inspection of the remaining feline H7N2 viral proteins revealed an absence of the most prominent amino acid changes known to facilitate adaptation to mammals, such as PB2–627K (*16*). These data thus suggest the 2016 feline H7N2 subtype viruses are avian-derived influenza viruses of low pathogenicity in avian and mammalian species.

### Replication of Feline and Avian H7N2 Subtype Viruses in Cultured Cells

**Figure 2:**
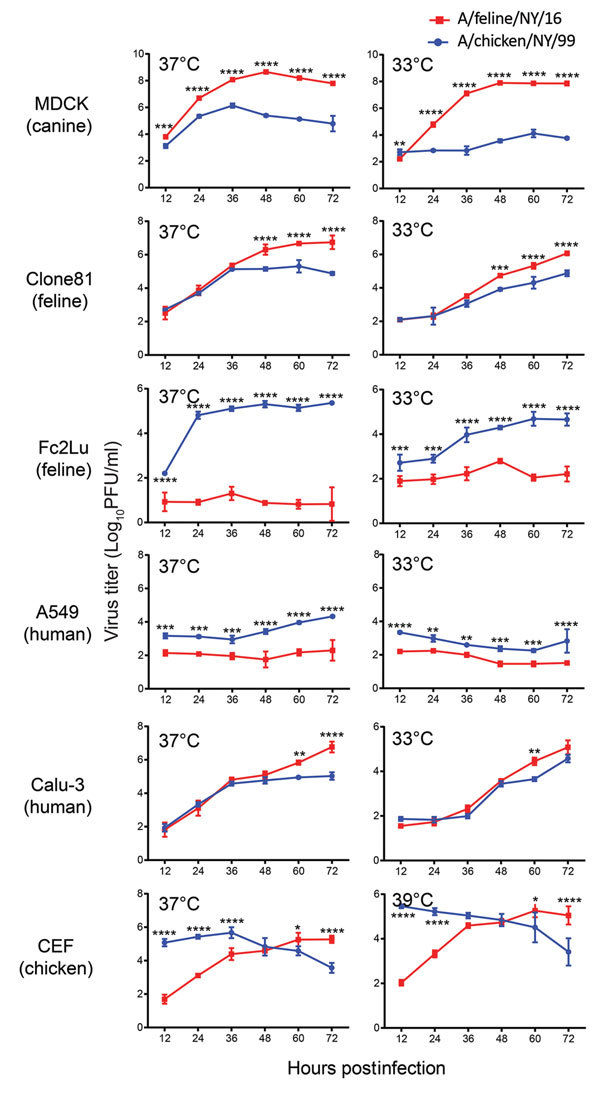
Growth properties of A/feline/NY/16 and A/chicken/NY/99 viruses in mammalian and avian cells at different temperatures. Cells were infected with viruses at a multiplicity of infection of 0.005 and incubated at 33° and 37°C (or at 37°C and 39°C for avian CEF cells). Supernatants were harvested at the indicated time points. Virus titers were determined by use of plaque assays in Madin-Darby canine kidney (MDCK) cells. A549, human lung carcinoma epithelial cells; Clone81, cat kidney fibroblast cells; Fc2Lu, cat lung cells; CEF, chicken embryo fibroblast cells. The species from which the cell lines are derived are shown. The values presented are the averages of 3 independent experiments ±standard deviation. Statistical significance was determined as described in the Technical Appendix. *, p<0.05; **, p<0.01; ***, p<0.001; ****, p<0.0001.

To characterize the replicative ability of the 2016 feline H7N2 viruses in cultured cells, we compared A/feline/NY/16 (which encodes the consensus amino acid sequence of the 5 isolates) with a closely related 1999 avian influenza virus, A/chicken/NY/22409–4/1999 (H7N2, A/chicken/NY/99) (Figure 1 and Technical Appendix Figures 3–9), which was isolated from a chicken in a live-bird market in New York state in 1999 (*8*). There are a total of 97 aa differences between A/feline/NY/16 and A/chicken/NY/99 viruses (12 aa differences in polymerase basic 2 (PB2), 7 in polymerase basic 1 (PB1), 12 in polymerase acidic (PA), 27 in hemagglutinin (HA), 8 in nucleoprotein (NP), 11 in neuraminidase (NA), 7 in matrix protein 1 (M1), 4 in matrix protein 2 (M2), and 9 in nonstructural protein 1 (NS1). Canine, human, feline, and chicken cells were infected at a multiplicity of infection of 0.005 at temperatures mimicking those of the upper and lower respiratory tract of the respective species (i.e., 37°C and 39°C for chicken cells; 33°C and 37°C for the remaining cells) (Figure 2). In canine MDCK, feline Clone81, and human Calu-3 cells, A/feline/NY/16 replicated at least as efficiently as A/chicken/NY/99 virus, while both viruses replicated to low titers in human A549 cells. Of note, A/feline/NY/16 virus replicated less efficiently than A/chicken/NY/99 virus in feline lung Fc2Lu cells. In chicken embryo fibroblast cells, A/feline/NY/16 virus replicated more slowly than A/chicken/NY/99 virus at early time points and reached its highest titers at later time points. When we compared virus growth at the 2 temperatures tested (i.e., 37°C and 39°C for chicken cells; 33°C and 37°C for the remaining cells), we observed similar trends (for example, in MDCK cells, A/feline/NY/16 replicated more efficiently than A/chicken/NY/99 at both temperatures tested).

### Replication and Pathogenicity of Feline and Avian H7N2 Subtype Viruses in Mice

To assess the replication of A/feline/NY/16 and A/chicken/NY/99 viruses in mice, 3 mice per group were inoculated intranasally with 10-fold dilutions of viruses, and their bodyweight and morbidity and mortality were monitored daily for 14 days. Mice infected with A/feline/NY/16 virus did not experience weight loss or signs of disease, whereas infection with 10^6^ PFU of A/chicken/NY/99 virus caused severe weight loss and required euthanasia (Technical Appendix Figure 10).

A/feline/NY/16 replicated efficiently in the nasal turbinates and less efficiently in the lungs of infected animals (Technical Appendix Figure 11); no virus was isolated from the other organs tested (i.e., brains, kidneys, livers, and spleens; data not shown). A/chicken/NY/99 replicated more efficiently in the lungs than in the nasal turbinates, consistent with immunohistochemistry analyses that detected A/feline/NY/16 virus antigens mainly in the upper respiratory organs of infected mice, whereas A/chicken/NY/99 virus antigens were detected more frequently in the lower respiratory organs (Technical Appendix Figure 12).

### Replication and Pathogenicity of Feline and Avian H7N2 Subtype Viruses in Ferrets

Ferrets intranasally infected with 10^6^ PFU of A/feline/NY/16 or A/chicken/NY/99 virus did not lose bodyweight (Technical Appendix Figure 13) but 2 of the ferrets infected with A/chicken/NY/99 virus had high fevers on day 1 postinfection. Both viruses replicated efficiently in the nasal turbinates and were also isolated from the trachea and lungs of some animals (Table 2), consistent with similar antigen distributions for both viruses (Technical Appendix Figure 14). No viruses were isolated from any of the other organs tested.

**Table 2:** Virus titers in organs of ferrets and cats infected with A/feline/NY/16 or A/chicken/NY/99 influenza viruses*

Species	Virus	Days post­infection	Animal ID no.	Virus titers in organs of infected animals (log_10_(PFU/g))					
Nasal turbinates	Trachea	Lung	Small intestine	Colon	Other organs†
Ferret	A/feline/NY/16	3	1	4.4	3.3	4.7	–	–	–
			2	5.2	–	2.4	–	–	–
			3	5.4	–	–	–	–	–
		6	4	3.1	–	–	–	–	–
			5	5.8	–	–	–	–	–
			6	6.0	–	–	–	–	–
	A/chicken/NY/99	3	7	5.9	3.3	–	–	–	–
			8	6.0	–	–	–	–	–
			9	6.6	3.4	–	–	–	–
		6	10	4.2	–	–	–	–	–
			11	4.5	–	5.7	–	–	–
			12	4.4	–	–	–	–	–
Cat	A/feline/ NY/16	3	1	3.9	4.8	–	–	–	–
			2	4.1	6.6	5.8	–	–	–
			3	6.9	7.0	5.8	–	–	–
		6	4	6.3	6.2	6.1	–	2.3	–
			5	7.7	7.8	4.7	–	–	–
			6	5.9	6.2	6.7	3.8	–	–
	A/chicken/NY/99	3	7	6.4	5.8	3.9	–	–	–
			8	2.0	–	–	–	–	–
			9	6.1	–	–	4.9	–	–
		6	10	6.4	–	5.0	–	–	–
			11	4.6	–	–	–	–	–
			12	6.7	4.0	–	–	–	–

*–, no virus detected.  †Brain, spleen, kidneys, liver, and pancreas.

### Replication and Pathogenicity of Feline and Avian H7N2 Subtype Viruses in Cats

**Figure 3:**
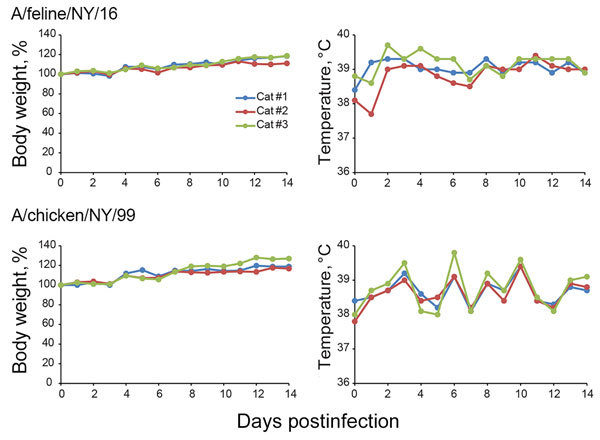
Body weight and temperature changes in cats infected with A/feline/NY/16 and A/chicken/NY/99 viruses. Three cats per group were infected intranasally with 10^6^ PFU of viruses and monitored for bodyweight and temperature changes.

The infection of ≈500 cats with H7N2 subtype viruses in animal shelters in New York in December 2016 suggested efficient replication of these viruses in felines. However, it was unclear whether these viruses were restricted to the respiratory organs or caused systemic infection. Cats intranasally infected with 10^6^ PFU of A/feline/NY/16 or A/chicken/NY/99 did not lose bodyweight (Figure 3); however, fever was detected in 1 animal infected with A/feline/NY/16, and 1 infected with A/chicken/NY/99 virus; and a different animal infected with A/feline/NY/16 sneezed intensely on day 3 postinfection, but recovered fully.

**Figure 4:**
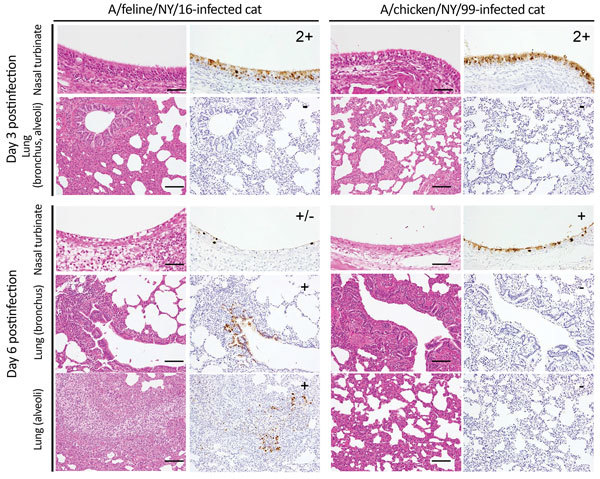
Immunohistochemistry findings in infected cats. Shown are representative sections of nasal turbinates and lungs of cats infected with the indicated viruses on days 3 and 6 postinfection. Three cats per group were infected intranasally with 10^6^ PFU of virus, and tissues were collected on days 3 and 6 postinfection. Type A influenza virus nucleoprotein (NP) was detected by a mouse monoclonal antibody to this protein. For nasal turbinate sections, -: no NP-positive cells, +/−: NP-positive cells detected in 1–2 focal regions, +: NP-positive cells detected in more than two focal regions, 2+: NP-positive cells in large regions. For bronchus and alveolar sections, -: no NP-positive cells, +/−: Up to 5 NP-positive cells, +: Six or more NP-positive cells. NP-positive cells were detected in focal, but not in diffuse bronchial and alveolar sections. For all analyses, the entire sections were evaluated. Scale bars, 50 μm (nasal turbinates), 100 μm (lung).

A/feline/NY/16 virus replicated efficiently in the nasal turbinates, trachea, and lungs of infected cats (with the exception of 1 cat with a virus-negative lung sample on day 3 postinfection; Table 2). We isolated A/chicken/NY/99 virus mostly from nasal turbinates, with limited replication in the trachea and lung. These findings are consistent with the detection of A/feline/NY/16 antigen in both the upper and lower respiratory organs of infected cats, whereas A/chicken/NY/99 antigen was detected mainly in the nasal turbinates (Figure 4). A/feline/NY/16 and A/chicken/NY/99 viruses were also isolated from the jejunum or colon of some of the infected animals (Table 2), although viral antigen was not detected in the intestines of cats infected with A/chicken/NY/99 or A/feline/NY/16 virus. These results demonstrate that the feline H7N2 virus replicates efficiently in the upper and lower respiratory tract of cats, reflecting adaptation of the virus to its new host.

**Figure 5:**
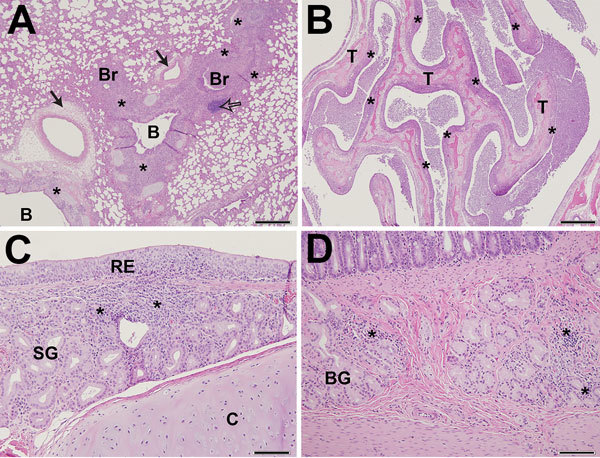
Pathology findings in cats infected with A/feline/NY/16 influenza A(H7N2) virus on day 6 postinfection, New York, NY, USA. A) In lungs, moderately severe histopathologic changes are present in the lower airways. The lamina propria of bronchi (B) and bronchioles (Br) and the surrounding interstitium are infiltrated by numerous histiocytes, lymphocytes, and plasma cells (*), which also extend into and expand neighboring alveolar septa. The infiltrates extend into and expand nearby alveolar septa. The lumina of bronchioles are filled with numerous foamy macrophages, viable and degenerating neutrophils, proteinaceous fluid, and sloughed respiratory epithelial cells. Hyperplasia of bronchiole-associated lymphoid tissue (open arrow) and perivascular edema (solid arrow) are present. Scale bar indicates 500 µm. B) In nasal cavities, copious amounts of exudate are present comprising numerous degenerating and necrotic neutrophils, cellular debris, proteinaceous fluid, and strands of mucin. The respiratory epithelium covering the nasal turbinates (T) is extensively eroded. The underlying lamina propria appears diffusely bluish-purple due to infiltration by moderate-to-large numbers of histiocytes, neutrophils, lymphocytes, and plasma cells (*). Scale bar indicates 500 µm. C) In the trachea, a locally extensive focus of inflammation is present in the tracheal wall. Moderate numbers of histiocytes, lymphocytes, and plasma cells, and a few neutrophils, infiltrate the respiratory epithelium (RE), lamina propria, and submucosa. Submucosal glands (SG) are surrounded by the inflammatory infiltrates and effaced in the areas of heaviest infiltration (*). Tracheal cartilage (C). Scale bar indicates 100 µm. D) In the duodenum, inflammatory cell infiltrates (*) in the submucosa of the duodenum are present between and around Brunner’s glands (BG). Scale bar indicates 100 µm.

All cats infected with the A/feline/NY/16 virus exhibited histologic lesions in their nasal turbinates, tracheas, and lungs. Nasal turbinate pathology was moderate to severe in 5 of 6 cats with multifocal to diffuse distribution of lesions (Figure 5, panel A). The tracheas of these cats exhibited mild to moderate histopathology (Figure 5, panel B), whereas the lungs exhibited multifocal to coalescing histopathology centered mostly on the bronchioles, with 3 of 6 cats possessing moderately severe lesions (Figure 5, panel C). Similar histopathological changes were found in cats infected with A/chicken/NY/99 virus. Appreciable histopathology was also noted in the small intestine (duodenum) of cats infected with A/feline/NY/16 and A/chicken/NY/99 viruses (Figure 5, panel D; cat ID nos. 1, 2, 4, 8, 10, and 12 in Table 2), although virus was detected in the intestines of only 3 cats (cat ID nos. 4, 6, and 9 in Table 2). The correlation between virus replication and histologic lesions in cat intestines is currently unknown.

### Transmission of Feline and Avian H7N2 Subtype Viruses in Ferrets and Cats

The fulminant spread of the feline H7N2 subtype viruses among cats, and the confirmed H7N2 virus infection of a veterinarian who treated the animals, indicate that these originally avian influenza viruses have the ability to transmit among mammals. To test the transmissibility of feline and avian H7N2 subtype viruses in ferrets, 3 animals per group (each placed in a separate cage) were infected intranasally with 10^6^ PFU (500 μL) of A/feline/NY/16 or A/chicken/NY/99 virus. One day later, we housed 1 naive ferret with each of the infected ferrets (direct contact transmission experiment), or placed naive ferrets in wireframe cages (within transmission isolators) ≈5 cm from the cages containing the infected ferrets as a respiratory droplet transmission experiment. We collected nasal wash samples from infected, contact, and exposed animals on day 1 after infection, contact, or exposure, and then every other day; we determined virus titers in nasal wash samples by use of plaque assays in MDCK cells (Table 3). In respiratory droplet transmission experiments, ferrets infected with A/feline/NY/16 or A/chicken/NY/99 virus secreted virus, but exposed animals were virus negative and did not seroconvert (Table 3). Among the direct contact animals, we detected virus in 1 ferret from the A/feline/NY/16-inoculated group and 2 from the A/chicken/NY/99-inoculated group; these 3 animals seroconverted, although the HI titer of 1 of the animals was low.

**Table 3:** Virus titers in nasal wash samples from ferret transmission studies*

Virus	Transmission mode	Pair	Action	Virus titers in nasal wash samples by days post-infection, exposure, or contact, log_10_(PFU/mL)						Seroconversion (HI titer)†
1	3	5	7	9	11
A/feline/ NY/16	Respiratory droplets	1	Infection	4.2	5.6	5.0	–	–	–	320
			Exposure	–	–	–	–	–	–	<10
		2	Infection	4.6	4.3	5.4	–	–	–	320
			Exposure	–	–	–	–	–	–	<10
		3	Infection	5.3	5.0	4.8	2.8	–	–	640
			Exposure	–	–	–	–	–	–	<10
	Direct contact	1	Infection	3.6	4.1	5.0	2.0	–	–	640
			Contact	–	–	–	–	–	–	<10
		2	Infection	5.5	5.1	4.3	1.3	–	–	320
			Contact	–	–	–	–	–	–	<10
		3	Infection	5.0	5.2	5.2	2.9	–	–	640
			Contact	–	–	4.2	5.3	4.6	–	320
A/chicken/ NY/99	Respiratory droplets	1	Infection	5.8	4.0	4.3	–	–	–	160
			Exposure	–	–	–	–	–	–	<10
		2	Infection	5.6	4.2	3.5	–	–	–	160
			Exposure	–	–	–	–	–	–	<10
		3	Infection	5.1	3.7	3.5	–	–	–	320
			Exposure	–	–	–	–	–	–	<10
	Direct contact	1	Infection	4.3	4.3	3.0	–	–	–	160
			Contact	–	–	3.8	4.3	3.4	–	160
		2	Infection	4.2	3.8	3.8	–	–	–	160
			Contact	–	–	2.1	–	–	–	10
		3	Infection	4.9	3.9	4.3	–	–	–	320
			Contact	–	–	–	–	–	–	10

*–, no virus detected. †Serum specimens were collected on day 18 after infection, exposure, or contact, and examined using an HI assay. The HI titer is the inverse of the highest dilution of serum that completely inhibited hemagglutination.

We conducted the transmission study in cats in the same way as the study in ferrets; we spaced cages 35 cm apart to prevent direct contact between the inoculated and exposed animals (Technical Appendix Figure 1, panel A). All infected cats secreted viruses for 5–7 days after infection and seroconverted, except for 1 cat infected with A/chicken/NY/99 virus, which seroconverted but did not shed virus (Table 4). We did not isolate A/chicken/NY/99 virus from contact or exposed animals, although these animals seroconverted (Table 4). Direct contact transmission of A/feline/NY/16 virus was detected in all 3 pairs of cats, with both seroconversion and virus isolation in 2 pairs (Table 4). Respiratory droplet transmission of A/feline/NY/16 occurred in 2 pairs of animals, with high virus titers detected in the nasal secretions of the exposed animals on days 9 and 11 postexposure, respectively; both of the exposed animals also seroconverted (Table 4). In the third transmission pair, the exposed animal did not shed virus or seroconvert. Taken together, we demonstrated that A/feline/NY/16 virus has the ability to transmit among cats via contact and respiratory droplets; the relative contribution of these modes of transmission to the H7N2 subtype virus outbreaks in cat shelters in New York is unknown.

**Table 4:** Virus titers in nasal swab samples from cat transmission studies*

Virus	Transmission mode	Pair	Action	Virus titers in nasal swab samples by days postinfection, exposure, or contact, log_10_(PFU/mL)							Sero-conversion (HI titer)†
1	3	5	7	9	11	13
A/feline/NY/16	Respiratory droplets	Pair 1	Infection	5.6	4.7	4.3	3.0	–	–	–	320
			Exposure	–	–	–	–	5.2	–	–	160
		Pair 2	Infection	4.5	2.7	5.0	4.6	–	–	–	320
			Exposure	–	–	–	–	–	5.4	–	80
		Pair 3	Infection	4.8	3.2	5.3	3.6	–	–	–	320
			Exposure	–	–	–	–	–	–	–	<10
	Direct contact	Pair 1	Infection	5.9	4.6	3.4	–	–	–	–	320
			Contact	–	–	2.0	5.4	–	–	–	320
		Pair 2	Infection	6.0	5.0	4.6	–	–	–	–	640
			Contact	–	–	–	–	–	–	–	80
		Pair 3	Infection	4.9	4.9	4.4	4.2	–	–	–	640
			Contact	–	5.2	5.4	5.2	–	–	–	160
A/chicken/NY/99	Respiratory droplets	Pair 1	Infection	4.0	3.7	4.7	–	–	–	–	320
			Exposure	–	–	–	–	–	–	–	20
		Pair 2	Infection	–	–	–	–	–	–	–	320
			Exposure	–	–	–	–	–	–	–	20
		Pair 3	Infection	2.6	1.6	2.3	–	–	–	–	80
			Exposure	–	–	–	–	–	–	–	160
	Direct contact	Pair 1	Infection	4.5	2.4	4.5	–	–	–	–	80
			Contact	–	–	–	–	–	–	–	160
		Pair 2	Infection	3.4	4.8	4.1	3.6	–	–	–	160
			Contact	–	–	–	–	–	–	–	40
		Pair 3	Infection	3.4	3.5	3.2	3.3	–	–	–	160
			Contact	–	–	–	–	–	–	–	20

*–, no virus detected. †Serum samples were collected on day 18 after infection, exposure, or contact, and examined by use of an HI assay. The HI titer is the inverse of the highest dilution of serum that completely inhibited hemagglutination.

### Receptor-Binding Specificity of Feline and Avian H7N2 Subtype Viruses

**Figure 6:**
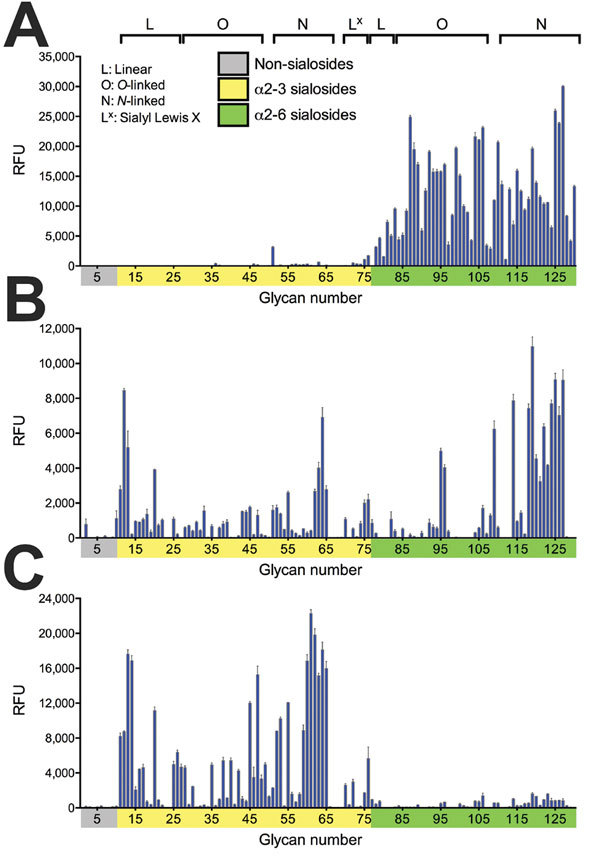
Receptor-binding specificities of influenza A viruses, New York, NY, USA. A) A representative human virus, A/Kawasaki/ 173-PR8(H1N1) is shown for comparison with B) the avian influenza A(H7N2) virus A/chicken/NY/99 and C) the feline influenza A(H7N2) virus A/feline/NY/16. Receptor-binding specificities of the avian and feline viruses were compared with those of the human virus in a glycan microarray containing α2,3- and α2,6-linked sialosides. Error bars represent SDs calculated from 4 replicate spots of each glycan. RFU, relative fluorescence units. A complete list of the glycans used is shown in Technical Appendix Table 2.

Avian influenza viruses isolated from their natural reservoir (i.e., wild aquatic birds) are often restricted in their ability to infect mammalian cells because of their preferential binding to α2,3-linked sialic acids, whereas most human influenza viruses preferentially bind to α2,6-linked sialic acids (*17*–*19*). We performed glycan array analysis with A/feline/NY/16, A/chicken/NY/99, and Kawasaki/173-PR8, a control virus possessing the HA and NA genes of the seasonal human A/Kawasaki/173/2001 (H1N1) virus and the remaining genes from A/PR/8/34 (H1N1) virus. As expected, Kawasaki/173-PR8 virus bound to α2,6-linked sialosides (Figure 6; Technical Appendix Table 2). A/chicken/NY/99 virus bound to both α2,6- and α2,3-linked sialosides, consistent with the dual avian/human receptor-binding specificity of influenza viruses isolated from land-based poultry (*1*). Of note, A/feline/NY/16 virus bound strongly to α2,3-linked sialosides (i.e., avian-type receptors) with negligible binding to human-type receptors.

**Figure 7:**
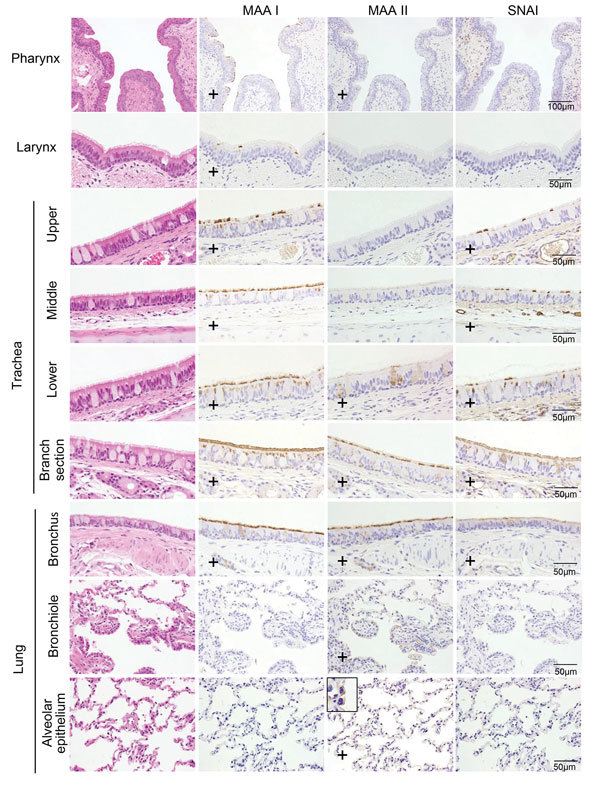
Distribution of α2,3- and α2,6-linked sialosides in the respiratory organs of a cat, New York, NY, USA. The α2,3- and α2,6-linked sialosides in the respiratory organs of a naïve cat were detected with biotinylated Maackia amurensis lectin I or II (MAA I, MAA II) or Sambucus nigra lectin (SNA I), respectively. Inset shows closer view of MAA III binding with alveolar epithelium in the lung. Plus signs (+) indicate that sialosides were detected. Scale bars indicate 50 μm.

Next, we examined the prevalence of α2,3- and α2,6-linked sialosides in the feline airway and intestines of an immunologically naive cat by using lectins that detect α2,3-linked (i.e., MAA I and MAA II) and α2,6-linked sialosides (i.e., SNAI). MAA I and MAIA II bound to epithelial cells throughout the feline airway, whereas SNA binding was detected only in the trachea and bronchus (Figure 7), consistent with the findings of other research groups (*20*–*22*). We did not detect sialosides in the cat intestine. The predominance of avian-type receptors in the upper respiratory tract of felines may have led to the selection of feline H7N2 virus HA proteins with preferential binding to α2,3-linked sialosides.

### Sensitivity to Neuraminidase Inhibitors

To test whether infections with the feline H7N2 viruses could be treated with neuraminidase (NA) inhibitors, we assessed the sensitivity of A/feline/NY/16 and A/chicken/NY/99 to several NA inhibitors (i.e., oseltamivir, zanamivir, and laninamivir) by determining the 50% inhibitory concentration (IC_50_) of the NA enzymatic activity. We used A/Anhui/1/2013 (H7N9) virus as an NA inhibitor–sensitive control and its NA inhibitor–resistant variant, A/Anhui/1/2013-NA-R294K, as an NA inhibitor–resistant control (Technical Appendix Table 3). A/feline/NY/16 and A/chicken/NY/99 were sensitive to all of the NA inhibitors tested (Technical Appendix Table 3), consistent with the absence of amino acid residues in the NA protein that are known to confer resistance to NA inhibitors. Hence, NA inhibitors could be used to treat persons infected with feline H7N2 subtype viruses.

## Discussion

In our study, we demonstrated that a feline H7N2 subtype virus isolated during an outbreak in an animal shelter in New York in December 2016 replicated well in the respiratory organs of mice and ferrets but did not cause severe symptoms. The efficient replication of the feline H7N2 subtype viruses in the respiratory organs of several mammals, combined with the ability of these viruses to transmit among cats (albeit inefficiently) and to infect 1 person, suggest that these viruses could pose a risk to human health. Close contacts between humans and their pets could lead to the transmission of the feline viruses to humans. To protect public health, shelter animals (where stress and limited space may facilitate virus spread) should be monitored closely for potential outbreaks of influenza viruses.

Our findings of mild disease in mice and ferrets are consistent with the recent report by Belser et al. (*7*) who studied the H7N2 subtype virus isolated from an infected veterinarian. We also assessed feline H7N2 virulence in cats and detected efficient virus replication in both the upper and lower respiratory organs of infected animals, whereas an avian H7N2 subtype virus was detected mainly in the nasal turbinates.

Belser et al. (*7*) reported that intranasal or aerosol infection of ferrets with the H7N2 virus isolated from the infected veterinarian did not result in the seroconversion of co-housed or exposed animals, although nasal wash samples from some of the co-housed ferrets contained low titers of virus; these findings may suggest limited virus transmission that was insufficient to establish a productive infection. In contrast, we detected feline H7N2 virus transmission to co-housed ferrets in 1 of 3 pairs tested; this difference may be explained by the amino acid differences in the PA, HA, and NA proteins of the feline and human H7N2 isolates (Technical Appendix Table 4) or by the small number of animals used in these studies. We also performed transmission studies in cats and detected feline H7N2 subtype virus transmission via direct contact and respiratory droplets. However, the group size used is a potential limitation of our study.

Cats are not a major reservoir of influenza A viruses, but can be infected naturally or experimentally with influenza viruses of different subtypes (*23*). Serologic surveys suggest high and low rates of seroconversion to seasonal human and highly pathogenic avian influenza viruses, respectively. Natural infections most likely result from close contact with infected humans or animals, and most of these infections appear to be self-limiting.

Few cases of human infections with influenza viruses of the H7 subtype were reported until 2013, and they typically caused mild illness; however, infection of a veterinarian with a highly pathogenic avian H7N7 virus had fatal consequences (*24*,*25*). Since 2013, influenza viruses of the H7N9 subtype have caused more than 1,300 laboratory-confirmed infections in humans, with a case-fatality rate of ≈30%. Although the current H7N9 and feline H7N2 subtype viruses do not exclusively bind to human-type receptors and do not transmit efficiently among humans, the spread and biologic properties of these viruses should be monitored carefully.

Technical Appendix Additional information from the study of feline influenza A(H7N2) virus isolated in New York.
