# Supplementary material for: Characterization of a Feline Influenza A(H7N2) Virus
Source: Emerg Infect Dis. 2018 Jan;24(1):75–86. doi: 10.3201/eid2401.171240 (PMC5749472; doi:10.3201/eid2401.171240)
Supplement: Technical Appendix — Additional information from the study of feline influenza A(H7N2) virus isolated in New York. [file 17-1240-Techapp-s1.pdf]

Article DOI: https://doi.org/10.3201/eid2401.171240

Characterization of a Feline Influenza A(H7N2) Virus

Technical Appendix

Supplementary Methods

Cells and Viruses

Madin-Darby canine kidney (MDCK) cells (obtained from ATCC) were maintained in Eagle's minimal essential medium (MEM) containing 5% newborn calf serum and antimicrobial drugs. Human lung carcinoma epithelial A549 cells were propagated in a 1:1 mixture of Dulbecco's modified Eagle's medium (DMEM) and Ham's F12 medium containing 10% fetal calf serum (FCS) with antimicrobial drugs. Human airway epithelial cells (Calu-3, obtained from Raymond Pickles, University of North Carolina, Chapel Hill, NC, USA) were cultured in DMEM/F12 medium supplemented with 10% FCS and antimicrobial drugs. Chicken embryo fibroblast (CEF) cells were prepared from 10-day-old chicken embryos and cultured in DMEM with 10% FCS and antimicrobial drugs. Cat kidney fibroblast Clone81 (ECACC 90031403) and cat lung Fc2Lu (ECACC 90112712) cells were purchased from the European Collection of Authenticated Cell Cultures (ECACC). Clone81 cells were cultured in DMEM with 10% FCS and antimicrobial drugs. Fc2Lu cells were maintained in MEM with 1% non-essential amino acids (NEAA), 10% FCS, and antimicrobial drugs. All cells were maintained at 37 °C with 5% CO_2 unless otherwise stated.

The viral genomic sequences of the 5 feline H7N2 viruses have been deposited in GenBank under the following accession numbers: A/feline/New York/WVDL-3/2016: MF978390–MF978397; A/feline/New York/WVDL-9/2016: MF978398–MF978405; A/feline/New York/WVDL-14/2016: MF978406–MF978413; A/feline/New York/WVDL-16/2016: MF978414–MF978421; A/feline/New York/WVDL-20/2016: MF978422–MF978429. The sequences of the HA, NA, M, and NS segments of A/chicken/NY/22409–4/1999 virus were available in GenBank (accession nos. AY240896, AY254122, AY241605, and AY241644, respectively) (*1*). We (re)sequenced all 8 viral segments and deposited the sequences of the PB2,

PB1, PA, and NP segments in GenBank under accession nos. MF988320–MF988323. The sequences of the HA and NA segments differed from AY240896 and AY254122 at the nucleotide, but not at the amino acid level, and were deposited in GenBank under accession nos. MF988323 and MF98825, respectively. The sequences of the M and NS segments were identical to AY241605 and AY241644, respectively, and therefore were not submitted to GenBank.

Negative Staining

MDCK cells were infected with A/feline/NY/16 and cultured in 1× MEM containing 0.3% bovine serum albumin and trypsin treated with L-1-tosylamide-2-phenylethyl chloromethyl ketone at 37°C. Forty-eight hours later, the supernatants were harvested and cell debris was removed by centrifugation at 1,750 x *g*. The virion-containing supernatants were adsorbed to Formvar-coated copper mesh grids, negatively stained with 2% phosphotungstic acid solution, and air dried. Digital images of virions were taken with a Tecnai F20 electron microscope (FEI, Tokyo, Japan) at 200 kV.

Animal Experiments

All experiments with mice, ferrets, and cats were performed in accordance with the guidelines set by the Institutional Animal Care and Use Committee at the University of Wisconsin–Madison, which also approved the protocols used (protocol numbers V00806 and V01190). The facilities where this research was conducted are fully accredited by the Association for the Assessment and Accreditation of Laboratory Animal Care International. The animal experiments described in this study were not designed to generate datasets for statistical analysis; hence, the sample size was small and randomization and blinding were not performed.

Immunohistochemistry

Tissues excised from animal organs preserved in 10% phosphate-buffered formalin were processed for paraffin embedding and cut into 5- and 3-μm-thick sections for hematoxylin and eosin staining and immunohistological staining, respectively. One section from each tissue sample was stained using a standard hematoxylin and eosin procedure; another sample was processed for immunohistological staining with a mouse monoclonal or rabbit polyclonal antibody for type A influenza nucleoprotein antigen (prepared in our laboratory) that reacts comparably with all of the viruses used in this study. Specific antigen–antibody reactions were visualized with 3,3′- diaminobenzidine tetrahydrochloride staining by using the DAKO LSAB2

system (Agilent, Santa Clara, CA, USA). Our negative controls (not shown) included sections from mock-infected animals. As a positive control (also not shown), we used formalin-fixed, paraffin-embedded lung sections from humans infected with seasonal influenza viruses.

Detection of α2,3- and α2,6-linked Sialosides in Cat Organs

To detect α2,3- and α2,6-linked sialosides (*2–4*), the tissues of a naive cat were fixed in 4% paraformaldehyde–phosphate-buffered saline (PBS) and embedded in paraffin. The paraffin blocks were cut into 3-μm-thick sections and mounted on silane-coated glass slides. The sections were pretreated with 0.05% trypsin (Difco Laboratories, Detroit, MI, USA) at 37°C for 15 min and then with 0.3% hydrogen peroxide at room temperature for 30 min. They were then incubated at 4°C overnight with biotin-conjugated Sambucus nigra lectin I (SNA I; EY Laboratories, San Mateo, CA, USA) to detect α2,6-linked sialosides, or with biotinylated-Maackia amurenis lectin I and II (MAAI and II; Vector Laboratories, Burlingame, CA, USA) to detect α2,3- linked sialosides. After being washed, the sections were incubated with horseradish peroxidase- conjugated streptavidin and visualized by staining with 3,3-diaminobenzidine (DAB).

Neuraminidase Inhibition Assay

Diluted viruses were mixed with different concentrations of oseltamivir carboxylate (the active form of oseltamivir), zanamivir, or laninamivir (all obtained from Daiichi Sankyo Co., Ltd, Tokyo, Japan) (*5*,*6*). Samples were incubated for 30 min at 37°C, followed by the addition of methylumbelliferyl-N-acetylneuraminic acid (Sigma, St Louis, MO) as a fluorescent substrate (*7*,*8*). After incubation for 1 h at 37°C, the reaction was stopped with the addition of sodium hydroxide in 80% ethanol. The fluorescence of the solution was measured at an excitation wavelength of 360 nm and an emission wavelength of 465 nm, and the 50% inhibitory concentration (IC_{50}) was calculated.

Glycan Array Analysis

Glycan array analysis was performed on a glass slide microarray containing 6 replicates of 130 diverse sialic acid-containing glycans, including terminal sequences and intact N-linked and O-linked glycans found on mammalian and avian glycoproteins and glycolipids (*9*). Viruses were amplified in MDCK cells. Supernatants collected from infected cells were centrifuged at 1,462 × *g* for 30 min to remove cell debris. Viruses were inactivated by mixing the supernatants

with 0.1% β-propiolactone (final concentration). Virus supernatant was laid over a cushion of 30% sucrose in PBS, ultracentrifuged at 76,755 × *g* for 2 h at 4°C, and then resuspended in PBS for storage at −80°C. Virus samples (equivalent of 128 hemagglutination units) were incubated on the array surface for 1 h at room temperature, and labeled with mouse monoclonal anti-H7/H1 IgG and goat anti-mouse IgG-Alex Fluor 488 antibodies for sequential 1-hour incubations. Slide scanning to detect virus bound to glycans was conducted using an Innoscan1100AL (Innopsys, Carbonne, France) fluorescent microarray scanner. Fluorescent signal intensity was measured using Mapix (Innopsys, Carbonne, France) and mean intensity minus mean background of 4 replicate spots was calculated. A complete list of the glycans on the array is presented in Technical Appendix, Table 2.

Hemagglutination Inhibition (HI) Assay

To detect hemagglutination inhibition (HI) activity (https://www.cdc.gov/flu/professionals/laboratory/antigenic.htm), serum samples were treated with receptor-destroying enzyme (Denka Seiken Co., Ltd., Tokyo, Japan) at 37°C for 16–20 hours, followed by receptor-destroying enzyme inactivation at 56°C for 30–60 min. The treated sera were serially diluted 2-fold with PBS in 96-well U-bottom microtiter plates (Thermo Scientific, Rochester, NY, USA) and mixed with the amount of virus equivalent to eight hemagglutination units, followed by incubation at room temperature (25°C) for 30 min. After 50 μL of 0.5% turkey erythrocytes was added to the mixtures, they were gently mixed and incubated at room temperature for a further 45 min. HI titers are expressed as the inverse of the highest antibody dilution that inhibited hemagglutination.

Statistical Analyses

We compared the values obtained for each strain, using a 2-way ANOVA, and creating a matrix of contrasts to compare each time-point separately. We then adjusted the p values by using Holm’s method to account for family-wise errors; we considered the differences significant if we obtained p values <0.05.

Phylogenetic Analysis

Phylogenetic analyses were carried out for selected avian and human influenza A viruses representing major lineages. The evolutionary history was inferred using the neighbor-joining method (*10*). The optimal trees were selected and the percentages of replicate trees in which the

associated taxa clustered together in the bootstrap test (500 replicates) (*11*) were identified. The trees were drawn to scale, with branch lengths in the same units as those of the evolutionary distances used to infer the phylogenetic tree. The evolutionary distances were computed using the Tamura 3-parameter method (*12*) and are in the units of the number of base substitutions per site. Codon positions included were 1st + 2nd + 3rd + Noncoding. All positions containing gaps and missing data were eliminated. Evolutionary analyses were conducted in MEGA7 (*13*).

Biosafety and Biosecurity

All recombinant DNA protocols were approved by the University of Wisconsin–Madison's Institutional Biosafety Committee after risk assessments were conducted by the Office of Biologic Safety. In addition, the University of Wisconsin–Madison Biosecurity Task Force regularly reviews the research program and ongoing activities of the laboratory. The task force has a diverse skill set and provides support in the areas of biosafety, facilities, compliance, security, and health. Members of the Biosecurity Task Force are in frequent contact with the principal investigator and laboratory personnel to provide oversight and assure biosecurity. The H7N2 viruses used in this study are low pathogenicity avian viruses according to the definition by the US Department of Agriculture and experiments with these viruses can be conducted in Biosafety Level 2+ (BSL2+) containment. For animal experiments with the feline H7N2 viruses, staff wore personal protective equipment including disposable coveralls, double gloves, dedicated shoes with disposable shoe covers, and powered air-purifying respirators that HEPA filter the air for extra protection. Ferret studies were conducted in BSL3 containment. All personnel working in BSL3 containment complete rigorous biosafety, BSL3, and Select Agent (for the US laboratory) training before participating in research studies. The principal investigator participates in training sessions and emphasizes compliance to maintain safe operations and a responsible research environment. The laboratory occupational health plans are in compliance with the policies of the University of Wisconsin–Madison.

References

1. Spackman E, Senne DA, Davison S, Suarez DL. Sequence analysis of recent H7 avian influenza viruses associated with three different outbreaks in commercial poultry in the United States. J Virol. 2003;77:13399–402. PubMed http://dx.doi.org/10.1128/JVI.77.24.13399-13402.2003

2. Shi Y, Wu Y, Zhang W, Qi J, Gao GF. Enabling the 'host jump': structural determinants of receptor-binding specificity in influenza A viruses. Nat Rev Microbiol. 2014;12:822–31. PubMed http://dx.doi.org/10.1038/nrmicro3362

3. Stencel-Baerenwald JE, Reiss K, Reiter DM, Stehle T, Dermody TS. The sweet spot: defining virus-sialic acid interactions. Nat Rev Microbiol. 2014;12:739–49. PubMed http://dx.doi.org/10.1038/nrmicro3346

4. Nicholls JM, Chan RW, Russell RJ, Air GM, Peiris JS. Evolving complexities of influenza virus and its receptors. Trends Microbiol. 2008;16:149–57. PubMed http://dx.doi.org/10.1016/j.tim.2008.01.008

5. Yamashita M. Laninamivir and its prodrug, CS-8958: long-acting neuraminidase inhibitors for the treatment of influenza. Antivir Chem Chemother. 2010;21:71–84. PubMed http://dx.doi.org/10.3851/IMP1688

6. Kamali A, Holodniy M. Influenza treatment and prophylaxis with neuraminidase inhibitors: a review. Infect Drug Resist. 2013;6:187–98. PubMed

7. Gubareva LV, Webster RG, Hayden FG. Comparison of the activities of zanamivir, oseltamivir, and RWJ-270201 against clinical isolates of influenza virus and neuraminidase inhibitor-resistant variants. Antimicrob Agents Chemother. 2001;45:3403–8. PubMed http://dx.doi.org/10.1128/AAC.45.12.3403-3408.2001

8. Kiso M, Kubo S, Ozawa M, Le QM, Nidom CA, Yamashita M, et al. Efficacy of the new neuraminidase inhibitor CS-8958 against H5N1 influenza viruses. PLoS Pathog. 2010;6:e1000786. PubMed http://dx.doi.org/10.1371/journal.ppat.1000786

9. Peng W, de Vries RP, Grant OC, Thompson AJ, McBride R, Tsogtbaatar B, et al. Recent H3N2 viruses have evolved specificity for extended, branched human-type receptors, conferring potential for increased avidity. Cell Host Microbe. 2017;21:23–34. PubMed http://dx.doi.org/10.1016/j.chom.2016.11.004

10. Saitou N, Nei M. The neighbor-joining method: a new method for reconstructing phylogenetic trees. Mol Biol Evol. 1987;4:406–25. PubMed

11. Felsenstein J. Confidence limits on phylogenies: an approach using the bootstrap. Evolution. 1985;39:783–91. PubMed http://dx.doi.org/10.1111/j.1558-5646.1985.tb00420.x

12. Tamura K. Estimation of the number of nucleotide substitutions when there are strong transition-transversion and G+C-content biases. Mol Biol Evol. 1992;9:678–87. PubMed

13. Kumar S, Stecher G, Tamura K. MEGA7: Molecular Evolutionary Genetics Analysis version 7.0 for bigger datasets. Mol Biol Evol. 2016;33:1870–4. PubMed http://dx.doi.org/10.1093/molbev/msw054

14. Kiso M, Iwatsuki-Horimoto K, Yamayoshi S, Uraki R, Ito M, Nakajima N, et al. Emergence of oseltamivir-resistant h7n9 influenza viruses in immunosuppressed cynomolgus macaques. J Infect Dis. 2017;216:582–93. PubMed http://dx.doi.org/10.1093/infdis/jix296

Technical Appendix Table 1. Antigenic characterization of H7 viruses by use of monoclonal antibodies

		Hemagglutination inhibition (HI) titers*							
		Mouse monoclonal antibody against							
		HA from A/Seal/Mass/1/80 (H7N7)				HA from A/Netherlands/ 219/03 (H7N7)	HA from A/Anhui/1/2013 (H7N9)		
Virus	Subtype	46/6	55/3	58/2	8/4	10C6	2–20–20	3–7-19	19–17–20
A/feline/New York/WVDL-14/2016 (Feline/NY/16)	H7N2	<50	<50	<50	100	400	200	100	400
A/chicken/NY/22409–4/1999 (Chicken/NY/99)		<50	800	<50	<50	400	200	100	200
A/duck/Hong Kong/301/1978		<50	50	1600	<50	<50	<50	<50	<50
A/turkey/England/1963	H7N3	<50	3200	800	<50	<50	<50	<50	<50
A/turkey/Oregon/1971		<50	50	200	<50	<50	200	<50	400
A/turkey/Tennessee/1/ 1976		<50	<50	100	<50	<50	<50	<50	<50
A/chicken/Japan/1925	H7N7	<50	<50	400	<50	<50	<50	<50	<50
A/equine/Prague/1/1956		<50	<50	<50	<50	200	<50	<50	<50
A/equine/New Market/1/1977		<50	<50	<50	<50	1600	<50	<50	<50
A/seal/Massachusetts/1/ 1980		3200	6400	3200	800	800	800	200	800
A/duck/Taiwan/103/1993		<50	<50	400	<50	<50	<50	<50	<50
A/duck/Gunma/466/2011	H7N9	<50	100	1600	<50	<50	100	<50	200
A/Anhui/1/2013		1600	3200	800	<50	800	200	50	400

*Hemagglutination inhibition (HI) assays were carried out as follows: 2-fold serial dilutions of antibodies were mixed with the amount of virus equivalent to 8 hemagglutination units of virus in 96-well U-bottom microtiter plates, followed by incubation at room temperature for 60 min. After an equal volume of 0.5% chicken red blood cells was added, the mixtures were gently mixed and then incubated for a further 60 min at 4°C. HI titers were determined as the inverse of the highest antibody dilution that inhibited the hemagglutination.

Technical Appendix Table 2. List of glycans used for arrays

No	M#	S#	Common Name	Linkage	NeuAC/NeuGc(A/B), both -C	Structure
1	**M040**	**M040**	Galβ(1–4)-GlcNAcβ-ethyl-NH2	–	–	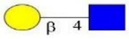
2	M221	WJ-5–149–1	Galβ(1-4)GlcNAcβ(1-3)Galβ(1-3)GalNAca-Thr-NH2	–	–	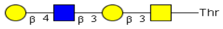
3	M222	152Sp14	Galβ(1-4)GlcNAcβ(1-6)[Galβ(1-3)]-GalNAca-Thr-NH2	–	–	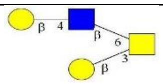
4	M223	144Sp14	Galβ(1-4)GlcNAcβ(1-3)GalNAca-Thr-NH2	–	–	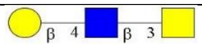
5	M224	21Sp14	Galβ(1-4)GlcNAcβ(1-3)[Galβ(1-4)GlcNAcβ(1-6)]-GalNAcα-Thr-NH2	–	–	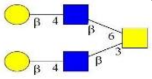
6	M225	119Sp14	Galβ(1-4)GlcNAcβ(1-6)GalNAcα-Thr-NH2	–	–	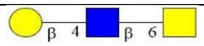
7	**M009**	**M009**	Galβ(1–4)-GlcNAcβ(1–2)-Manα(1-3)-[Galβ(1–4)-GlcNAcβ(1–2)-Manα(1–6)]-Manβ(1–4)-GlcNAcβ(1- 4)-GlcNAcβ-Asn-NH2	–	–	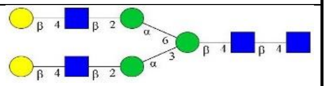
8	M226	375Sp22	Galβ(1-4)GlcNAcβ(1-2)Manα(1-3)[Galβ(1-4)GlcNAcβ(1-2)Manα(1-6)]-Manβ(1-4)GlcNAcβ(1-4)[Fucα(1-6)]-GlcNAcβ-Asn-Ser-Thr-NH2	–	–	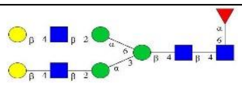

No	M#	S#	Common Name	Linkage	NeuAC/Neu Gc(A/B), both -C	Structure
9	M227	487Sp19	Galβ(1-4)GlcNAcβ(1-2)Manα(1-3){Galβ(1-4)GlcNAcβ(1-2)[Galβ(1-4)GlcNAcβ(1-2)]-Manα(1-6)}-Manβ(1-4)GlcNAcβ(1-4)GlcNAcβ-Asn-Lys-NH2	–	–	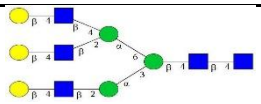
10	M228	517Sp	Galβ(1-4)GlcNAcβ(1-2)Manα(1-3){Galβ(1-4)GlcNAcβ(1-2)[Galβ(1-4)GlcNAcβ(1-2)]-Manα(1-6)}-Manβ(1-4)GlcNAcβ(1-4)[Fucα(1-6)]-GlcNAcβ-(Lys-Val-Ala)Asn-Lys-ThrNH2	–	–	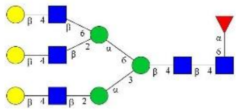
11	**M001**	**M001**	NeuAcα(2-3)Galβ(1-4)6-O-sulfoGlcNAcβ-propyl-NH2	3	A	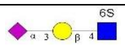
12	**M037**	**M037**	NeuAcα(2–3)-Galβ(1–4)-[Fucα(1–3)]-6-O-sulfo-GlcNAcβ-propyl-NH2	3	A	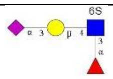
13	**M039**	**M039**	NeuAcα(2–3)-6-O-sulfo-Galβ(1–4)- GlcNAcβ-ethyl-NH2	3	A	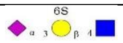
14	**M036**	**M036**	NeuAcα(2–3)-6-O-sulfo-Galβ(1–4)-[Fucα(1–3)]-GlcNAcβ-propyl-NH2	3	A	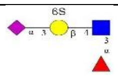
15	**M038**	**M038**	NeuAcα(2–3)-Galβ(1–3)-6-O-sulfo-GlcNAcβ-propyl-NH2	3	A	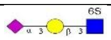

No	M#	S#	Common Name	Linkage	NeuAC/Neu Gc(A/B), both -C	Structure
16	**M011**	**M011**	NeuAca(2–3)- Galβ(1–4)- Glcβ-ethyl-NH2	3	A	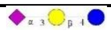
17	**M012**	**M012**	NeuAca(2–3)- Galβ(1–4)- GlcNAcβ-ethyl-NH2	3	A	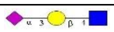
18	**M014**	**M014**	NeuAca(2–3)- Galβ(1–4)- GlcNAcβ(1- 3)-Galβ(1–4)-GlcNAcβ-ethyl-NH2	3	A	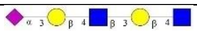
19	**M035**	**M035**	NeuAcα(2–3)- Galβ(1–4)- GlcNAcβ(1- 3)-Galβ(1–4)- GlcNAcβ(1–3)- Galβ(1- 4)-	3	A	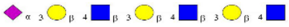
20	**M013**	**M013**	NeuAca(2–3)- GalNAcβ(1–4)- GlcNAcβ-ethyl-NH2	3	A	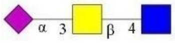
21	**M010**	**M010**	NeuAca(2–3)- Galβ(1–3)- GlcNAcβ-ethyl-NH2	3	A	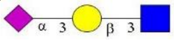
22	**M032**	**M032**	NeuAca(2–3)- Galβ(1–3)- GlcNAcβ(1- 3)-Galβ(1–4)-GlcNAcβ-ethyl-NH2	3	A	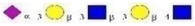
23	**M033**	**M033**	NeuAca(2–3)- Galβ(1–3)- GlcNAcβ(1- 3)-Galβ(1–3)-GlcNAcβ-ethyl-NH2	3	A	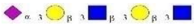
24	**M028**	**M028**	NeuAca(2–3)- Galβ(1–3)- GalNAcβ(1- 3)-Gala(1–4)- Galβ(1–4)- Glcβ-ethyl-	3	A	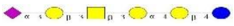
25	**M045**	**M045**	NeuAcα(2–3)- Galβ(1–3)- GalNAcα-Thr-NH2	3	A	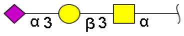

26	M120	WJ-6–121–1	3′ NeuAc LN Core 1 (1163)	3	A	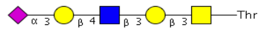
27	M128	WJ-6–153–1	3′ NeuAc DiLN Core 1 (1528)	3	A	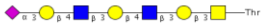
28	M153	WJ-8–145–1	3′ NeuAc TriLN Core 1 (1894)	3	A	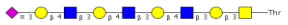
29	M142	WJ-8–101–1	3′ NeuAc TetraLN Core 1 (2259)	3	A	
30	M143	WJ-8–103–1	3′ NeuAc PentaLN Core 1 (2624)	3	A	
31	**M050**	**M050**	NeuAcα(2–3)-Galβ(1–4)-GlcNAcβ(1-6)-[Galβ(1–3)]-GalNAcα-Thr-NH_2	3	A	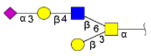
32	**M053**	**M053**	NeuAcα(2–3)-Galβ(1–4)-GlcNAcβ(1-3)-Galβ(1–4)-GlcNAcβ(1–6)-[Galβ(1-	3	A	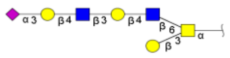
33	**M202**	WJ-9–41–1	3′ NeuAc TriLN Core 2 (1894)	3	A	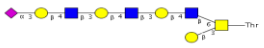
34	M152	WJ-8–141–1	3′ NeuAc TetraLN Core 2 (2259)	3	A	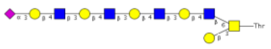
35	M149	WJ-8–131–1	3′ NeuAc PentaLN Core 2 (2624)	3	A	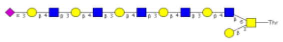
36	**M195**	WJ-9–13–1	3′ NeuAc TetraLN TriLN Core 2 (3645)	3	A	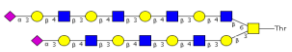

37	M156	WJ-8–155–1	3′ NeuAc PentaLN TetraLN Core 2 (4376)	3	A	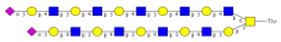
38	**M055**	**M055**	NeuAcα(2–3)-Galβ(1–4)-GlcNAcβ(1- 3)-GalNAcα-Thr-NH_2	3	A	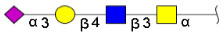
39	**M057**	**M057**	NeuAcα(2–3)-Galβ(1–4)-GlcNAcβ(1-3)-Galβ(1–4)-GlcNAcβ(1–3)-GalNAcα-Thr-NH_2	3	A	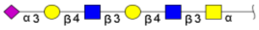
40	**M186**	WJ-8–99–1	3′ NeuAc TriLN Core 3 (1732)	3	A	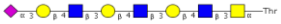
41	**M178**	WJ-8–83–1	3′ NeuAc TetraLN Core 3 (2097)	3	A	
42	**M177**	WJ-8–77–1	3′ NeuAc PentaLN Core 3 (2462)	3	A	
43	**M059**	**M059**	NeuAcα(2–3)-Galβ(1–4)-GlcNAcβ(1-3)-[NeuAcα(2–3)-Galβ(1–4)-GlcNAcβ(1–6)]-	3	A	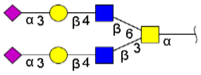
44	**M061**	**M061**	NeuAcα(2–3)-Galβ(1–4)-GlcNAcβ(1-3)-Galβ(1–4)-GlcNAcβ(1–3)-[NeuAcα(2–3)-	3	A	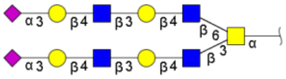
45	**M185**	WJ-8–97–1	3′ NeuAc TriLN Core4 (3118)	3	A	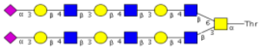
46	**M180**	WJ-8–87–1	3′ NeuAc TetraLN Core4 (3848)	3	A	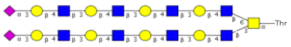
47	**M179**	WJ-8–85–1	3′ NeuAc PentaLN Core4 (4579)	3	A	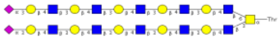

48	**M182**	WJ-8–91–1	3′ NeuAc TetraLN Core6 (2097)	3	A	
49	**M184**	WJ-8–95–1	3′ NeuAc PentaLN Core6 (2462)	3	A	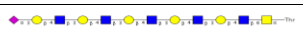
50	**M102**	WJ-10–71–1	3′ NeuAc LecLN I-Antigen(2104)	3	A	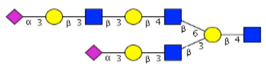
51	**M098**	WJ-10–61–1	3′ NeuAc TriLN I-Antigen (2856)	3	A	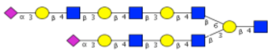
52	**M026**	**M026**	NeuAcα(2–3)-Galβ(1–4)-GlcNAcβ(1-2)-Manα(1–3)-[NeuAcα(2–3)-Galβ(1-	3	A	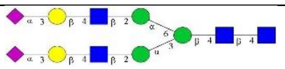
53	**M041**	**M041**	NeuAcα(2–3)-Galβ(1–4)-GlcNAcβ(1-3)-Galβ(1–4)-GlcNAcβ(1–2)-Manα(1–3)-	3	A	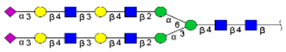
54	**M043**	**M043**	NeuAcα(2–3)-Galβ(1–4)-GlcNAcβ(1-3)-Galβ(1–4)-GlcNAcβ(1–3)-Galβ(1-	3	A	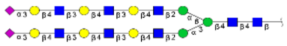
55	**M107**	WJ-5–21–1	3′ NeuAc DiLN Bi-(3594)	3	A	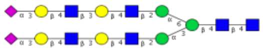
56	**M110**	WJ-5–35–1	3′ NeuAc TriLN Bi-(4324)	3	A	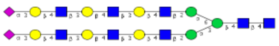
57	**M112**	WJ-5–39–1	3′ NeuAc TetraLN Bi-(4828)	3	A	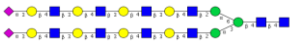
58	**M114**	WJ-5–45–1	3′ NeuAc PentaLN Bi-(5556)	3	A	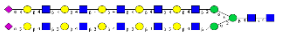

59	M132	WJ-6–41–1	3′ NeuAc TriLN Bi-CF(4470)	3	A	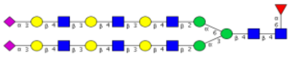
60	M122	WJ-6–13–1	3′ NeuAc TetraLN Bi-CF(5200)	3	A	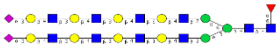
61	M118	WJ-6–117–1	3′ NeuAc DiLN Tri-(4615)	3	A	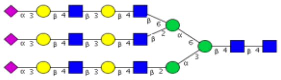
62	M119	WJ-6–119–1	3′ NeuAc TriLN Tri-(5716)	3	A	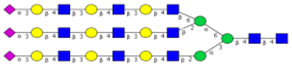
63	M141	WJ-7–47–1	3′ NeuAc TetraLN Tri-(6808)	3	A	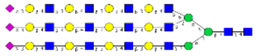
64	M125	WJ-6–149–1	3′ NeuAc DiLN Tri-CF(4761)	3	A	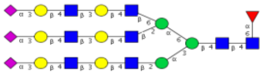
65	M127	WJ-6–151–1	3′ NeuAc TriLN Tri-CF(5858)	3	A	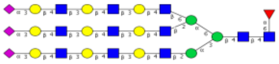
66	**M068**	701_WJ-10–91–1	Gn/3′SLN/3′SLN-TriN	3	A	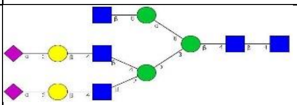
67	**M031**	**M031**	NeuAca(2–3)-[GalNAcβ(1–4)]-Galβ(1–4)-GlcNAcβ-ethyl-NH_2	3	A	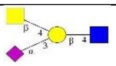
68	**M016**	**M016**	NeuAca(2–3)-[GalNAcβ(1–4)]-Galβ(1–4)-Glcβ-ethyl-NH_2	3	A	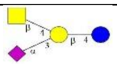
69	**M017**	**M017**	Galβ(1–3)-GalNAcβ(1–4)-[NeuAca(2–3)]-Galβ(1–4)-Glcβ-ethyl-NH_2	3	A	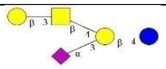

70	**M002**	**M002**	NeuAca(2–3)-Galβ(1–4)-[Fuca(1–3)]-GlcNAcβ-propyl-NH_2	3	A	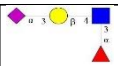
71	**M029**	**M029**	NeuAca(2–3)-Galβ(1–3)-[Fuca(1–4)]-GlcNAcβ(1–3)-Galβ(1–4)-[Fuca(1- 3)]-	3	A	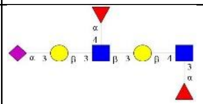
72	**M022**	**M022**	NeuAca(2–3)-Galβ(1–4)-[Fuca(1–3)]-GlcNAcβ(1–3)-Galβ(1–4)-[Fuca(1- 3)]-	3	A	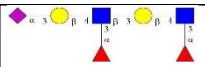
73	**M015**	**M015**	NeuAca(2–3)-Galβ(1–4)-[Fuca(1–3)]-GlcNAcβ(1–3)-Galβ(1–4)-[Fuca(1-3)]-GlcNAcβ(1–3)-	3	A	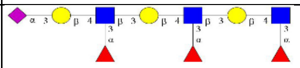
74	**M206**	WJ-9–7-1	3′ SLeX TriLN Core 1(2332)	3	A	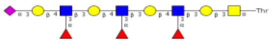
75	M147	WJ-8–127–1	3′ SLeX TriLN Core 3(2170)	3	A	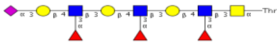
76	M146	WJ-8–125–1	3′ SLeX TriLN Core 4(3994)	3	A	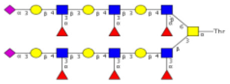
77	M219	WJ-119–1	NeuAca(2-3)Galβ(1-4)[Fuca(1-3)]-GlcNAcβ(1-2)Mana(1-3)[NeuAca(2-3)Galβ(1-4)[Fuca(1-3)]-GlcNAcβ(1-2)Mana(1-6)]-Manβ(1-4)GlcNAcβ(1-4)GlcNAcβ-(Lys-Val-Ala)Asn-(Lys-Thr)NH2	3	A	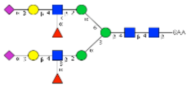
78	M215	WJ-12–79–1	NeuAc(2–6)-Galb(1–4)-(6S)GlcNacb-ethyl-NH2	6	A	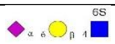
	**M003**	**M003**	NeuAca(2–6)-Galβ(1–4)-6-O-sulfo- GlcNAcβ-propyl-NH2	6	A	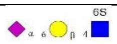

80	**M018**	**M018**	NeuAca(2–6)-Galβ(1–4)-Glcβ-ethyl-NH2	6	A	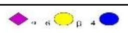
81	**M019**	**M019**	NeuAca(2–6)-Galβ(1–4)-GlcNAcβ-ethyl-NH2	6	A	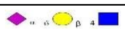
82	**M021**	**M021**	NeuAca(2–6)-Galβ(1–4)-GlcNAcβ(1- 3)-Galβ(1–4)-GlcNAcβ-ethyl-NH2	6	A	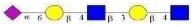
83	**M025**	**M025**	NeuAca(2–6)-Galβ(1–4)-GlcNAcβ(1-3)-Galβ(1–4)-GlcNAcβ(1–3)-Galβ(1- 4)-GlcNAcβ-	6	A	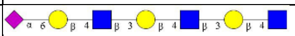
84	**M020**	**M020**	NeuAca(2–6)-GalNAcβ(1–4)- GlcNAcβ-ethyl-NH2	6	A	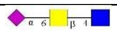
85	M121	WJ-6–123–1	6’ NeuAc LN Core 1 (1163)	6	A	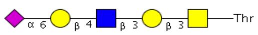
86	M129	WJ-6–155–1	6’ NeuAc DiLN Core 1 (1528)	6	A	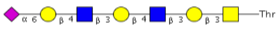
87	M154	WJ-8–147–1	6’ NeuAc TriLN Core 1 (1894)	6	A	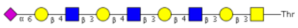
88	M135	WJ-7–149–1	6’ NeuAc TetraLN Core 1 (2259)	6	A	
89	M148	WJ-8–13–1/WJ-7–107-1	6’ NeuAc PentaLN Core 1 (2624)	6	A	
90	**M051**	**M051**	NeuAcα(2–6)-Galβ(1–4)-GlcNAcβ(1-6)-[Galβ(1–3)]-GalNAcα-Thr-NH$_2$	6	A	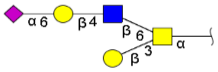

91	**M054**	**M054**	NeuAcα(2–6)-Galβ(1–4)-GlcNAcβ(1-3)-Galβ(1–4)-GlcNAcβ(1–6)-[Galβ(1-	6	A	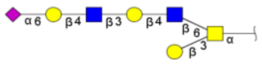
92	**M201**	WJ-9–39–1	6’ NeuAc TriLN Core 2 (1894)	6	A	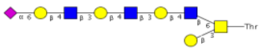
93	M159	WJ-8–23–1	6’ NeuAc TetraLN Core 2 (2259)	6	A	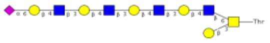
94	M157	WJ-8–17–1	6’ NeuAc PentaLN Core 2 (2624)	6	A	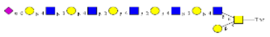
95	M163	WJ-8–33–1	6’ NeuAc TetraLN TriLN Core 2 (3645)	6	A	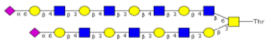

96	M161	WJ-8–29–1	6’ NeuAc PentaLN TetraLN Core 2 (4376)	6	A	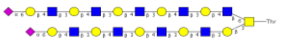
97	**M056**	**M056**	NeuAcα(2–6)-Galβ(1–4)-GlcNAcβ(1- 3)-GalNAcα-Thr-NH2	6	A	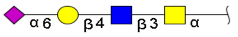
98	**M058**	**M058**	NeuAcα(2–6)-Galβ(1–4)-GlcNAcβ(1-3)-Galβ(1–4)-GlcNAcβ(1–3)-GalNAcα-Thr-NH$_2$	6	A	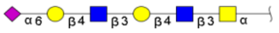
99	M172	WJ-8–65–1	6’ NeuAc TriLN Core 3 (1732)	6	A	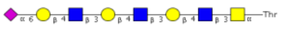
100	M166	WJ-8–49–1	6’ NeuAc TetraLN Core 3 (2097)	6	A	
101	M164	WJ-8–35–1	6’ NeuAc PentaLN Core 3 (2462)	6	A	

102	**M060**	**M060**	NeuAcα(2–6)-Galβ(1–4)-GlcNAcβ(1-3)-[NeuAcα(2–6)-Galβ(1–4)-GlcNAcβ(1–6)]-	6	A	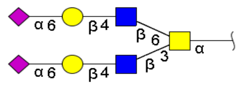
103	**M062**	**M062**	NeuAcα(2–6)-Galβ(1–4)-GlcNAcβ(1-3)-Galβ(1–4)-GlcNAcβ(1–3)-[NeuAcα(2–6)-	6	A	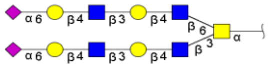
104	M174	WJ-8–73–1	6' NeuAc TriLN Core4 (3118)	6	A	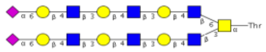
105	M170	WJ-8–61–1	6' NeuAc TetraLN Core4 (3848)	6	A	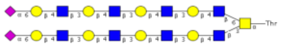
106	M168	WJ-8–57–1	6' NeuAc PentaLN Core4 (4579)	6	A	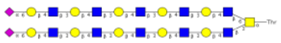
107	**M181**	WJ-8–89–1	6' NeuAc TetraLN Core6 (2097)	6	A	
108	**M183**	WJ-8–93–1	6' NeuAc PentaLN Core6 (2462)	6	A	
109	**M097**	WJ-10–59–1	6' NeuAc TriLN I-Antigen (2856)	6	A	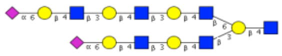
110	**M104**	WJ-10–77–1	6' NeuAc DiLN I-Antigen (2104)	6	A	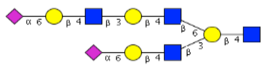
111	**M006**	**M006**	Galβ(1–4)-GlcNAcβ(1–2)-Manα(1-3)-[NeuAcα(2–6)-Galβ(1–4)-GlcNAcβ(1–2)-	6	A	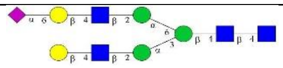
112	**M007**	**M007**	NeuAcα(2–6)-Galβ(1–4)-GlcNAcβ(1-2)-Manα(1–3)-[Galβ(1–4)-GlcNAcβ(1–2)-	6	A	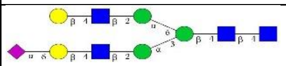

113	**M008**	**M008**	GlcNAcβ(1–2)-Manα(1–3)-[NeuAcα(2–6)-Galβ(1–4)-GlcNAcβ(1–2)-Manα(1–6)]-	6	A	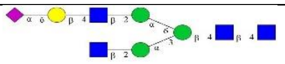
114	**M004**	**M004**	NeuAcα(2–6)-Galβ(1–4)-GlcNAcβ(1-2)-Manα(1–3)-[NeuAcα(2–6)-Galβ(1-	6	A	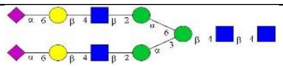
115	**M042**	**M042**	NeuAcα(2–6)-Galβ(1–4)-GlcNAcβ(1-3)-Galβ(1–4)-GlcNAcβ(1–2)-Manα(1–3)-[NeuAcα(2–6)-Galβ(1–4)-GlcNAcβ(1–3)-Galβ(1–4)-GlcNAcβ(1–2)-Manα(1–6)]-Manβ(1- 4)-GlcNAcβ(1–4)-GlcNAcβ-Asn- $^{NH}2$	6	A	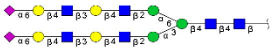
116	**M109**	WJ-5–33–1	6’ NeuAc DiLN Bi-(3594)	6	A	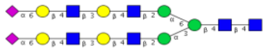
117	**M044**	**M044**	NeuAcα(2–6)-Galβ(1–4)-GlcNAcβ(1-3)-Galβ(1–4)-GlcNAcβ(1–3)-Galβ(1-	6	A	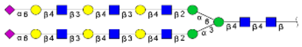
118	**M089**	JP-3–8-1	6’ NeuAc TriLN Bi-(4324)	6	A	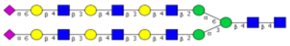
119	**M081**	JP-3–12–1	6’ NeuAc TetraLN Bi-(4828)	6	A	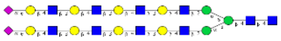
120	**M083**	JP-3–16–1	6’ NeuAc PentaLN Bi-(5556)	6	A	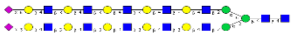
121	**M085**	JP-3–20–2	6’ NeuAc DiLN Bi-CF(3740)	6	A	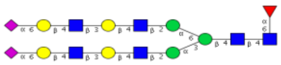
122	**M087**	JP-3–24–1	6’ NeuAc TriLN Bi-CF(4470)	6	A	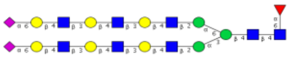

123	M131	WJ-6–25–1	6’ NeuAc TetraLN Bi-CF(5200)	6	A	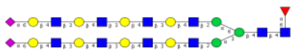
124	M123	WJ-6–133–1	6’ NeuAc DiLN Tri-(4615)	6	A	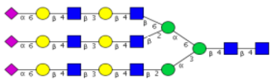
125	M134	WJ-7–13–1	6’ NeuAc DiLN Tri-CF(4761)	6	A	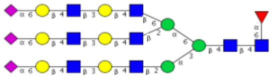
126	M136	WJ-7–15–1	6’ NeuAc TriLN Tri-CF(5858)	6	A	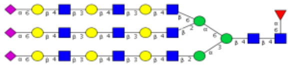
127	M138	WJ-7–35–1	6’ NeuAc TetraLN Tri-CF(6952)	6	A	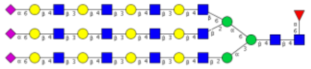
128	**M065**	112_WJ-10–147–1	LN/6'SLN/6'SLN-TriN	6	A	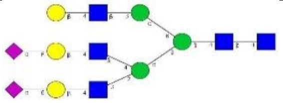
129	**M067**	128_WJ-10–149–1	6'SLN/LeX/LeX-TriN	6	A	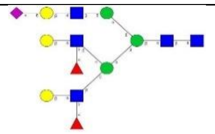
30	**M063**	047_WJ-10–145–1	6'SLNLN/LeX/LeX-TriN	6	A	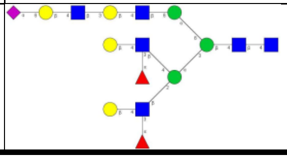

Technical Appendix Table 3. Virus sensitivity to NA inhibitors

	IC_{50} value*			
NA inhibitors	Chicken/NY/99	Feline/NY/16	A/Anhui/1/2013§ (H7N9)	A/Anhui/1/2013-NA-R294K¶ (H7N9)
Oseltamivir carboxylate†	1.6	1.0	3.6	64,000
Zanamivir	5.6	8.2	8.1	340
Laninamivir‡	15	17.5	3.4	210

*IC_{50} value: mean nmol/L of duplicate reactions.
†Oseltamivir carboxylate is the active form of oseltamivir.
‡Laninamivir is the active form of laninamivir octanoate.
§A/Anhui/1/2013 (H7N9): NA inhibitor-sensitive virus.
¶A/Anhui/1/2013-NA-R294K (H7N9): NA inhibitor-resistant virus (14).

Technical Appendix Table 4. Amino acid differences among A/feline/NY/16 virus and human H7N2 isolate (A/New York/108/2016)

Virus	Amino acid positions in the viral proteins					
	PA	HA			NA	
	57	9	127	156	40	362
A/feline/NY/16	Q	T	S	T	Y	R
A/New York/108/2016*	R	I	N	A	H	K

*The sequences were obtained from GISAID (accession nos. EPI944622–EPI944629). PA, polymerase; HA, hemagglutinin; NA, neuraminidase.

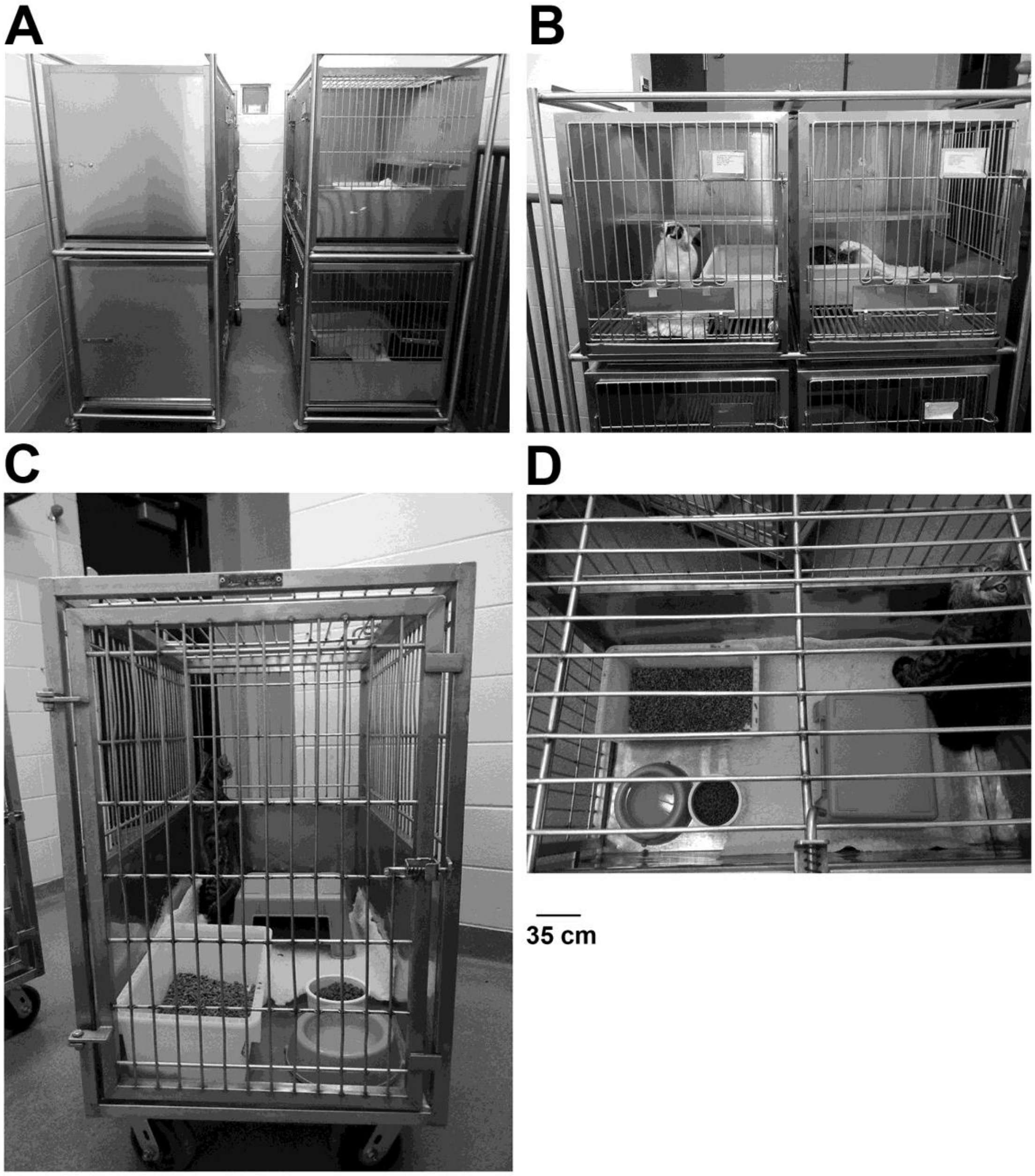

Technical Appendix Figure 1. Cage settings for virus transmission studies in cats. All cat transmission experiments were conducted at the Charmany Instructional Facility, School of Veterinary Medicine, University of Wisconsin–Madison, under controlled conditions of temperature and humidity. (A and B) Cages and racks used for respiratory droplet transmission studies. Cats were housed individually in regular cat cages. The two racks holding infected and naïve cats were spaced 35 cm apart to prevent direct and indirect contact between animals while allowing respiratory droplet transmission of influenza viruses. (C and D) Cages used for direct contact transmission studies. Large dog transporter cages with a perch/resting platform were used. One infected and one naïve cat were housed together in one cage.

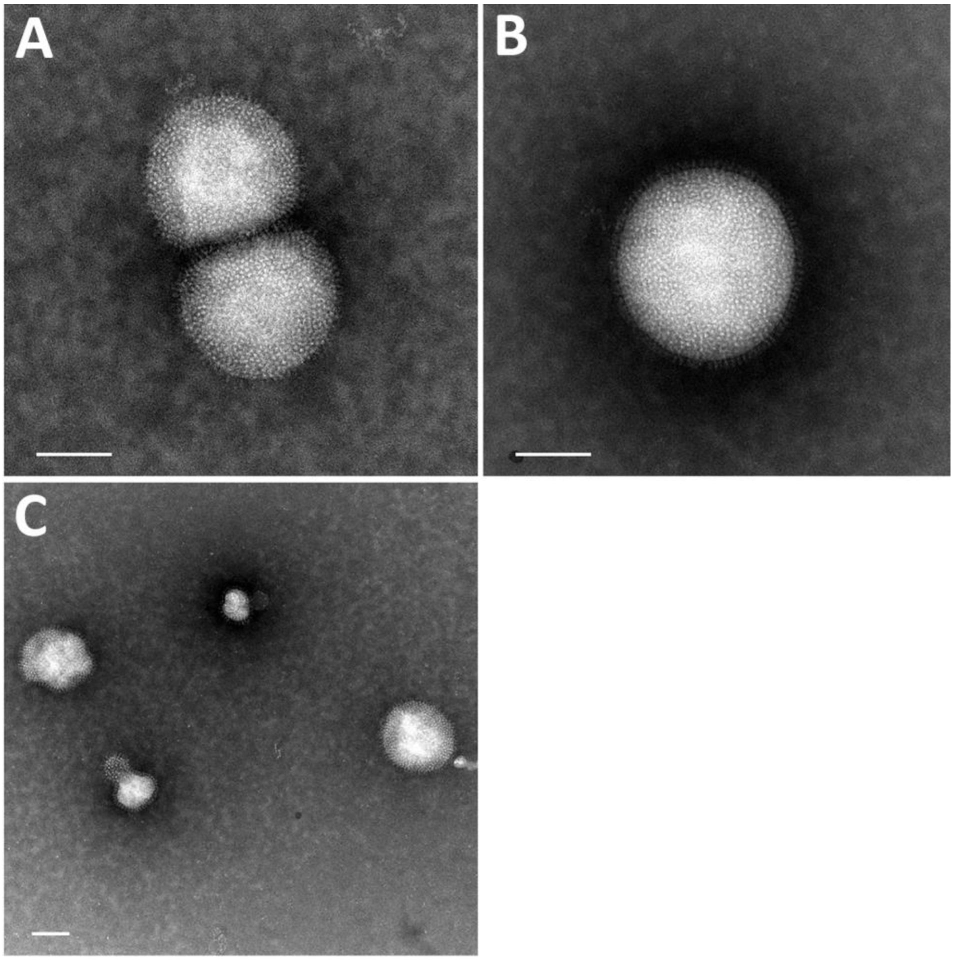

Technical Appendix Figure 2. Images of feline H7N2 virions observed by negative-staining electron microscopy. Virions negatively stained with 2% phosphotungstic acid solution were observed under an electron microscope. (A and B) Higher magnification of virus particles. (C) Lower magnification of virus particles. Scale bar = 100 nm.

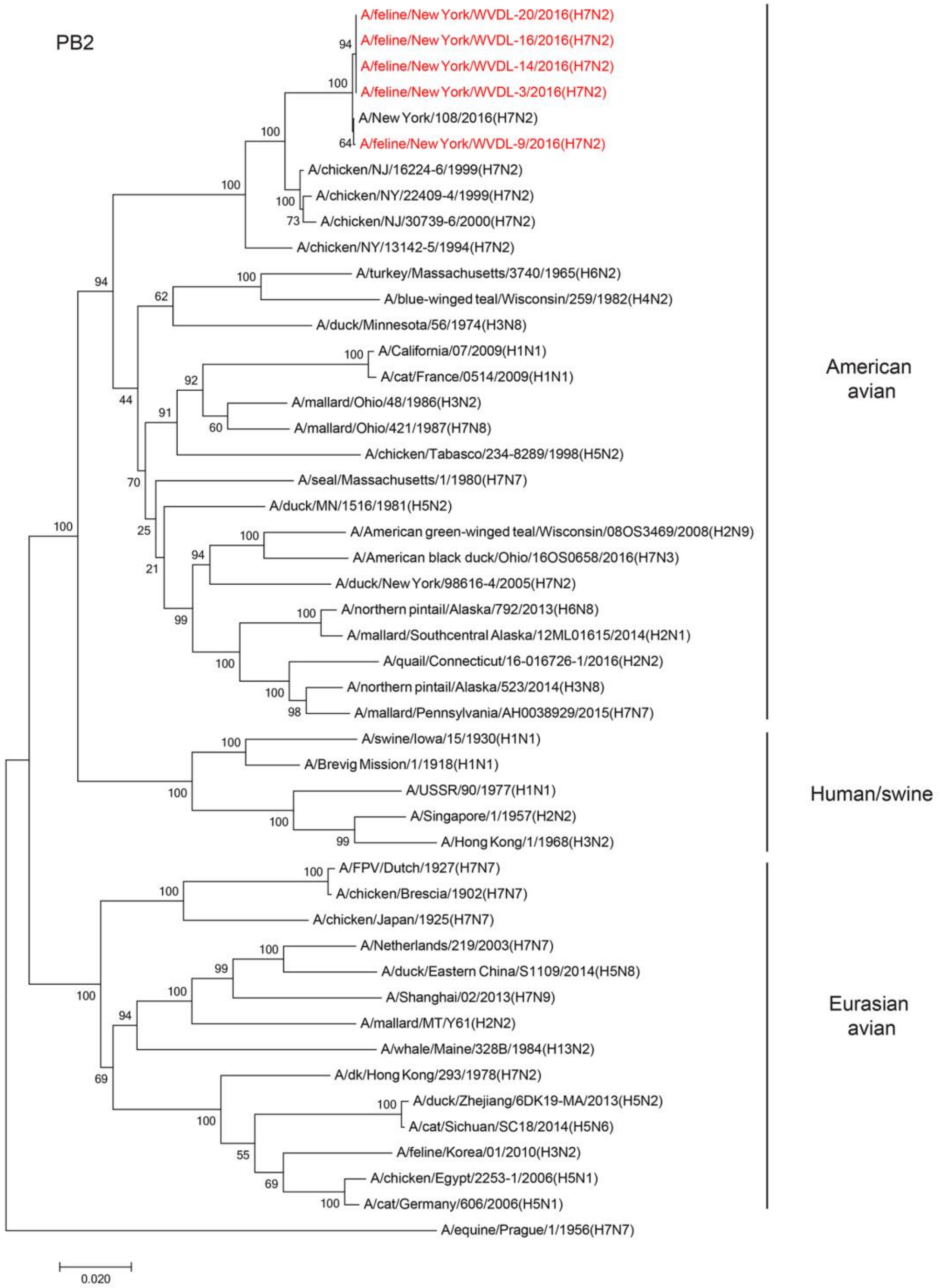

Technical Appendix Figure 3. Phylogenetic tree of influenza A viral PB2 segments. The optimal tree with the sum of branch length = 1.59092622 is shown. The analysis involved 48 nt sequences. The final dataset contained a total of 2,260 positions.

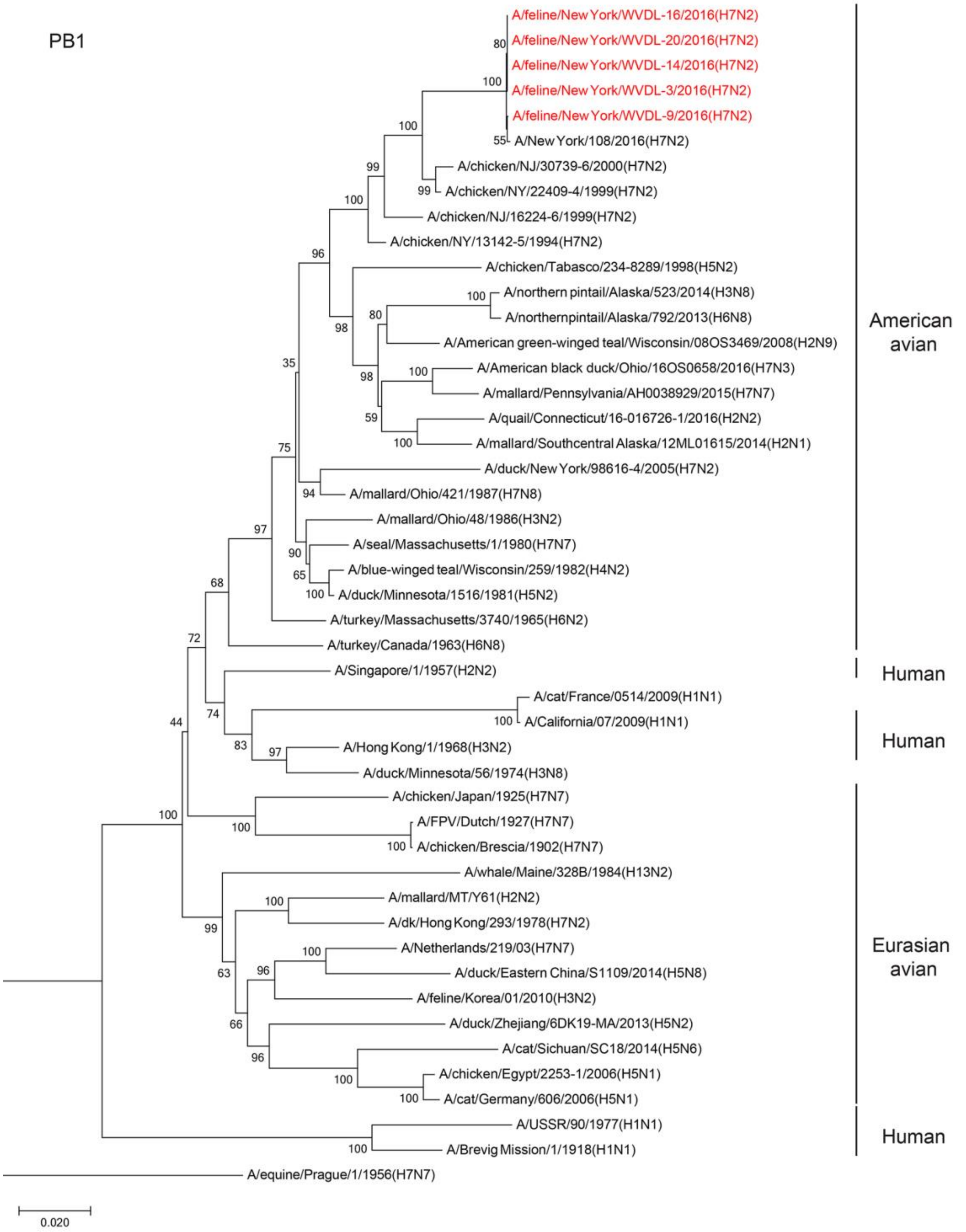

Technical Appendix Figure 4. Phylogenetic tree of influenza A viral PB1 segments. The optimal tree with the sum of branch length = 1.3928728 is shown. The analysis involved 47 nt sequences. The final dataset contained a total of 2,263 positions.

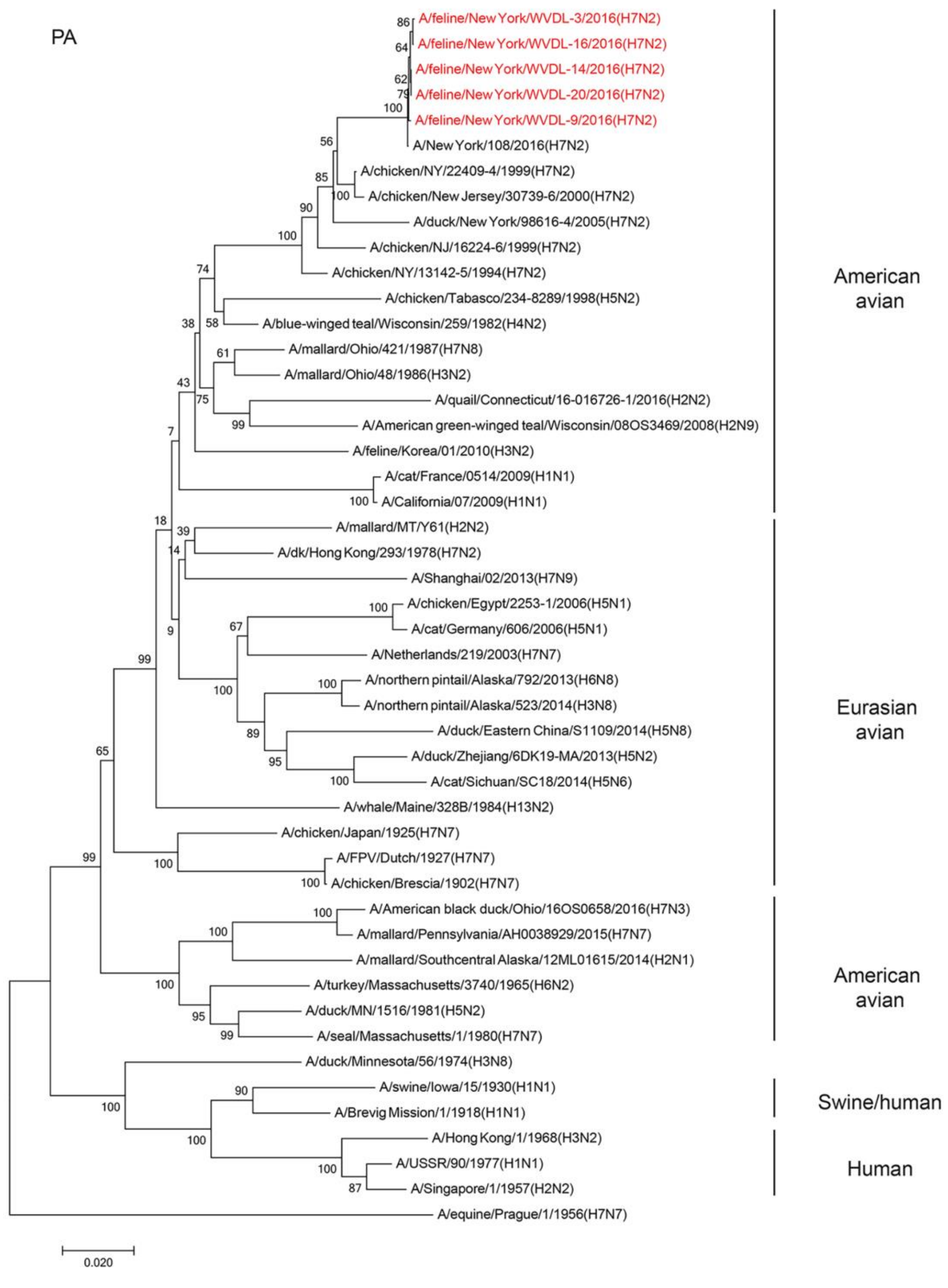

Technical Appendix Figure 5. Phylogenetic tree of influenza A viral PA segments. The optimal tree with the sum of branch length = 1.51709379 is shown. The analysis involved 48 nt sequences. The final dataset contained a total of 2,090 positions.

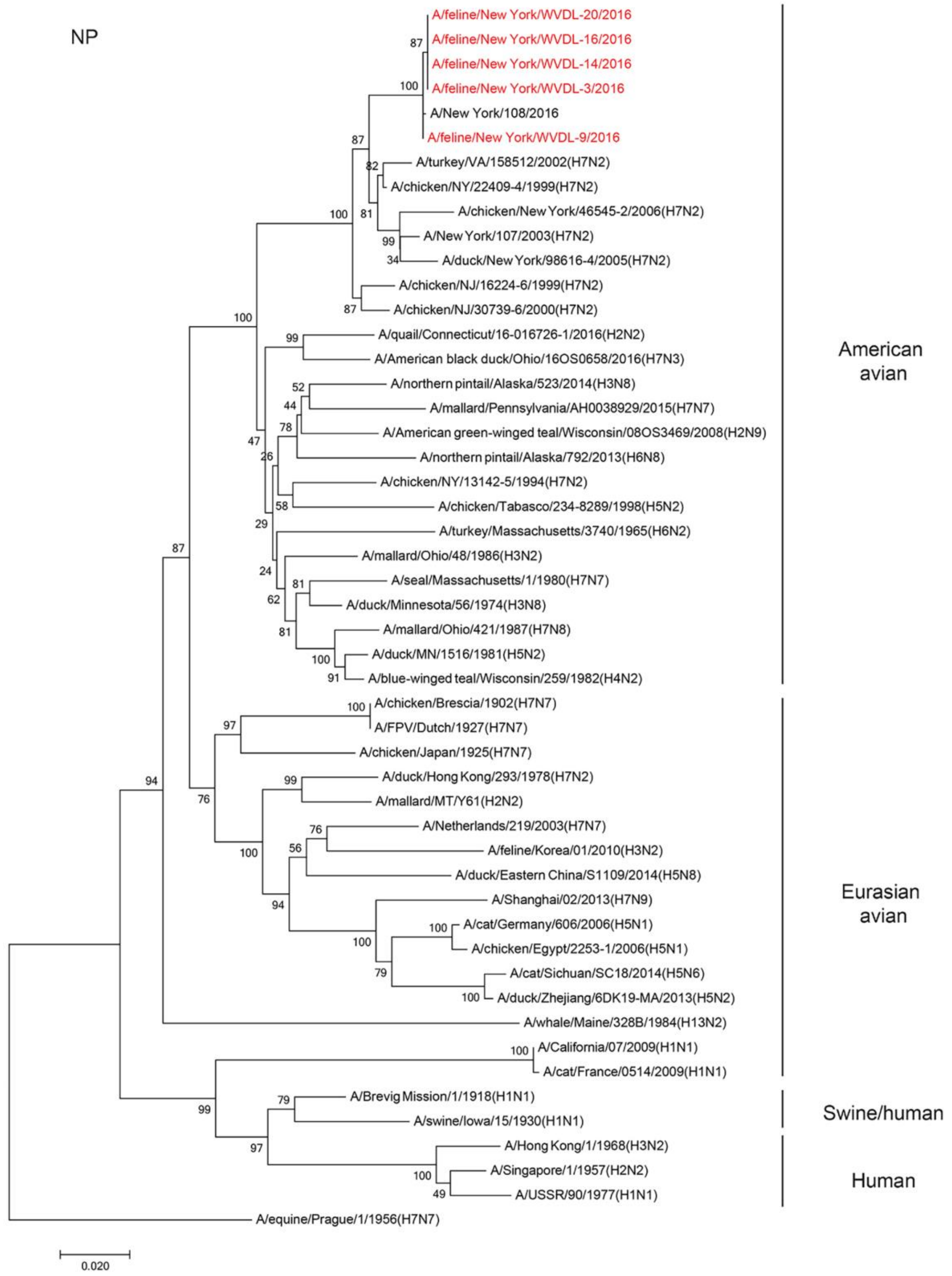

Technical Appendix Figure 6. Phylogenetic tree of influenza A viral NP segments. The optimal tree with the sum of branch length = 1.44906153 is shown. The analysis involved 50 nt sequences. The final dataset contained a total of 1,444 positions.

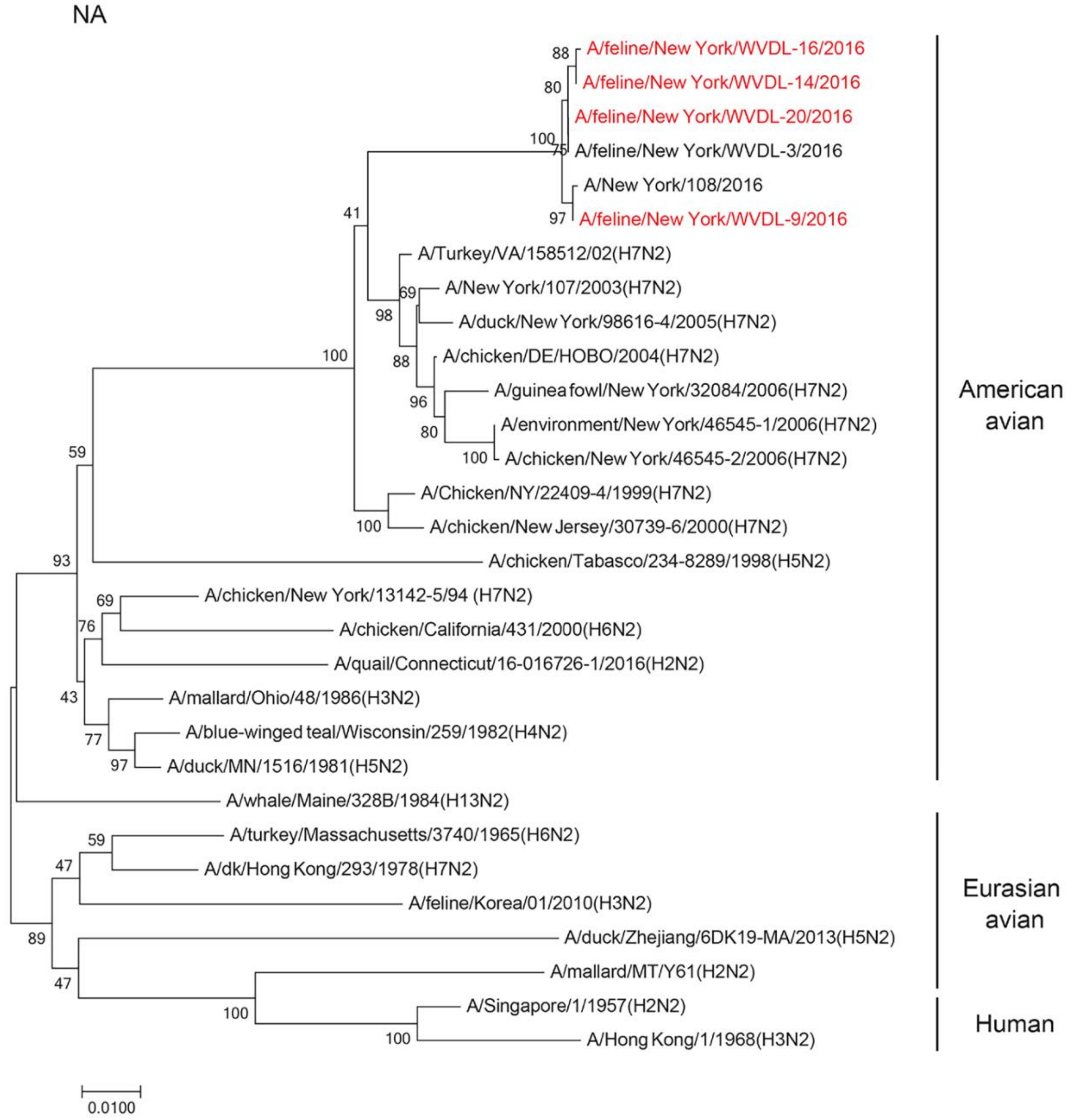

Technical Appendix Figure 7. Phylogenetic tree of influenza A viral NA segments. The optimal tree with the sum of branch length = 0.72173357 is shown. The analysis involved 31 nt sequences. The final dataset contained a total of 1,343 positions.

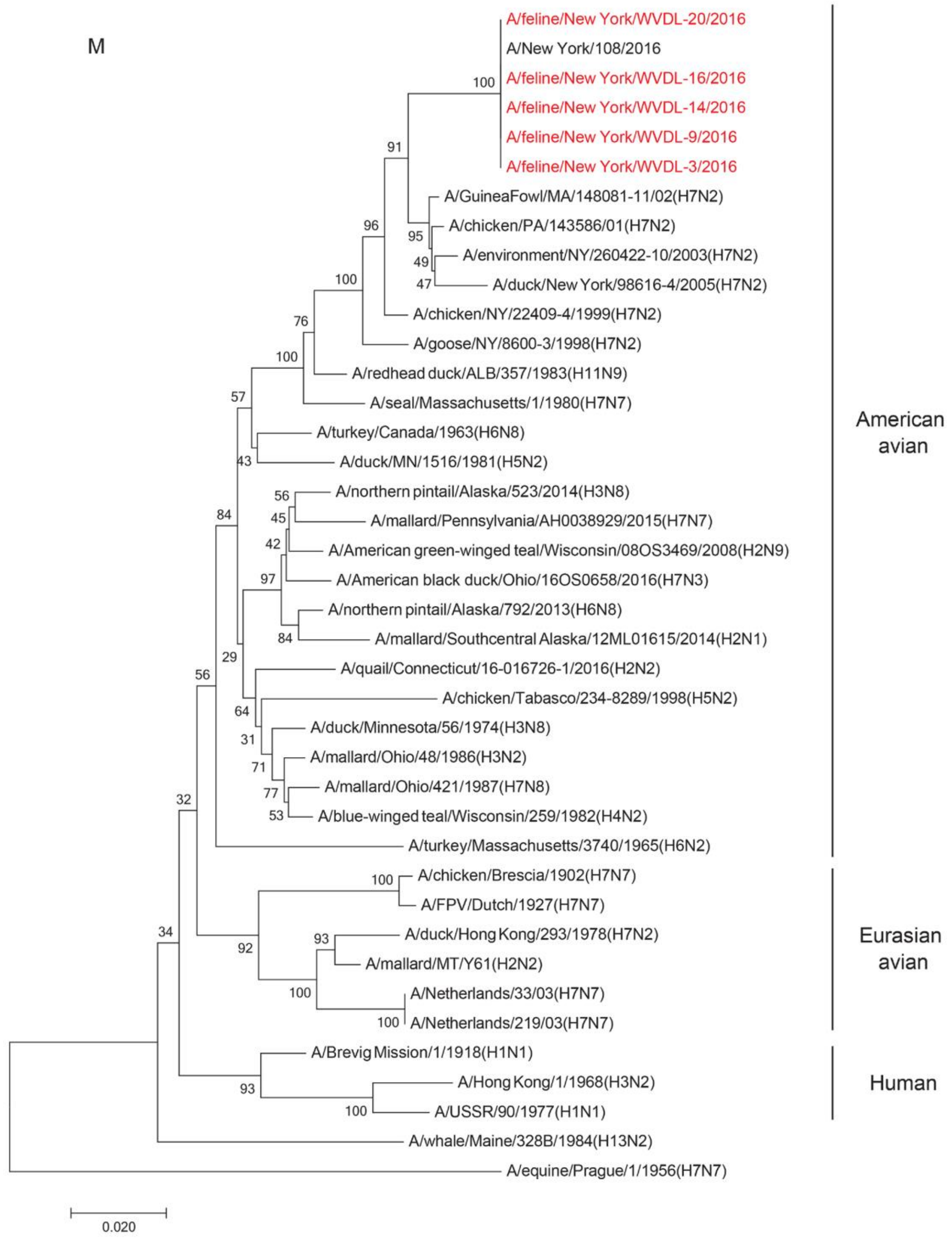

Technical Appendix Figure 8. Phylogenetic tree of influenza A viral M segments. The optimal tree with the sum of branch length = 0.72235656 is shown. The analysis involved 41 nt sequences. The final dataset contained a total of 971 positions.

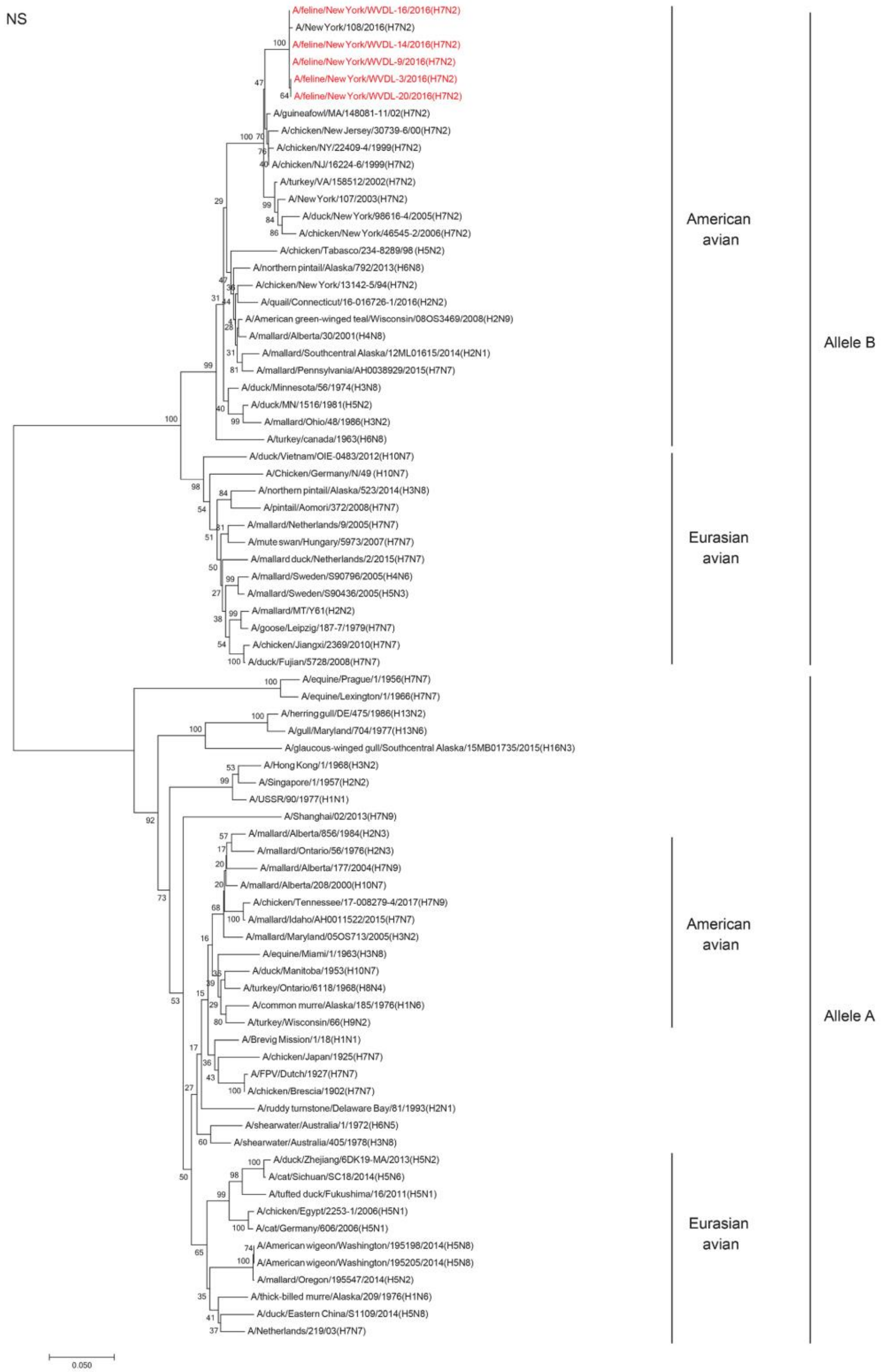

Technical Appendix Figure 9. Phylogenetic tree of influenza A viral NS segments. The optimal tree with the sum of branch length = 1.97636652 is shown. The analysis involved 79 nt sequences. The final dataset contained a total of 811 positions.

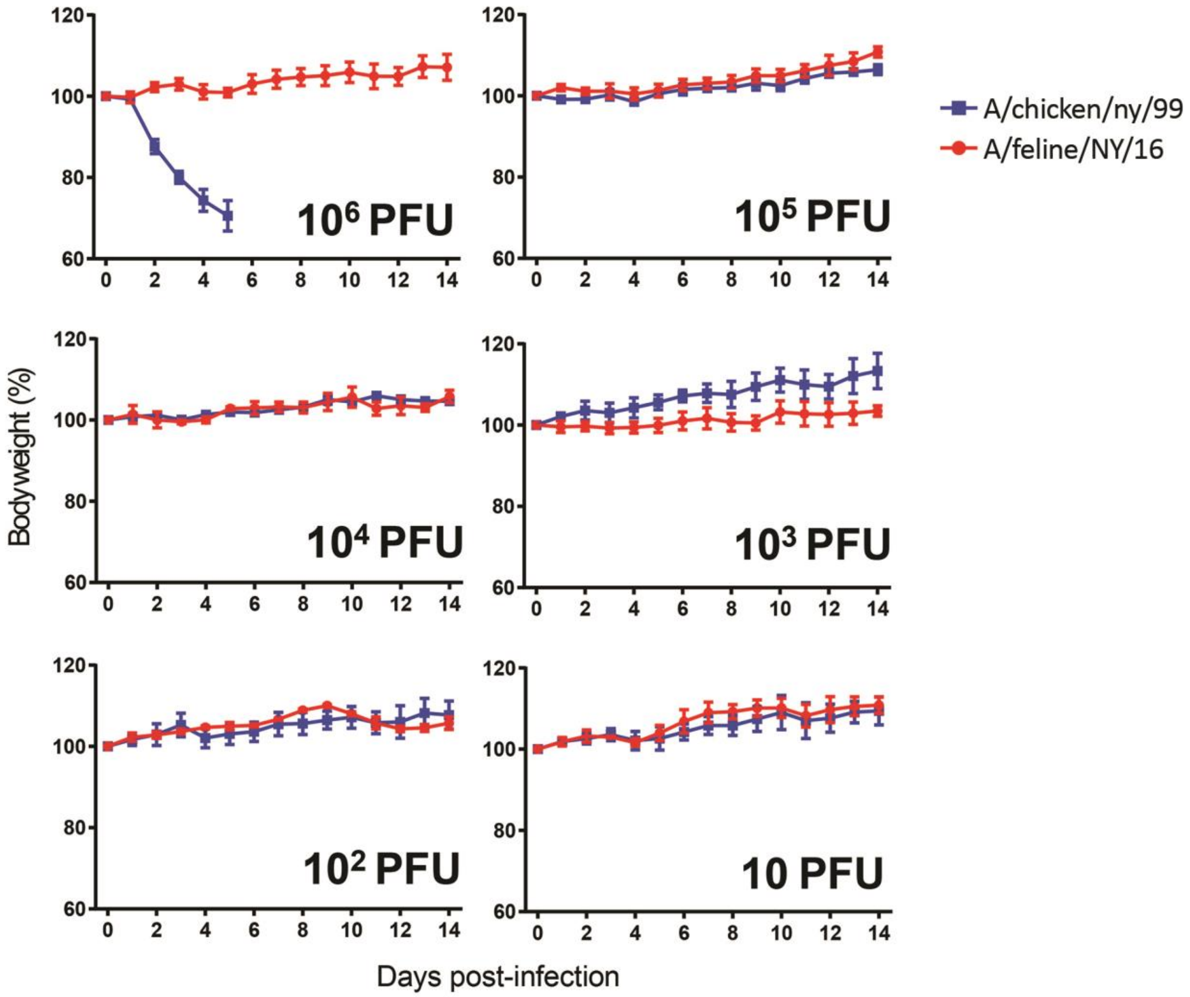

Technical Appendix Figure 10. Pathogenicity of A/feline/NY/16 and A/chicken/NY/99 viruses in mice. Bodyweight changes in mice infected with A/feline/NY/16 and A/chicken/NY/99 viruses. Three mice per group were infected intranasally with A/feline/NY/16 and A/chicken/NY/99 virus in amounts of 10–10^6 PFU. Bodyweight and morbidity and mortality were monitored daily for 14 days.

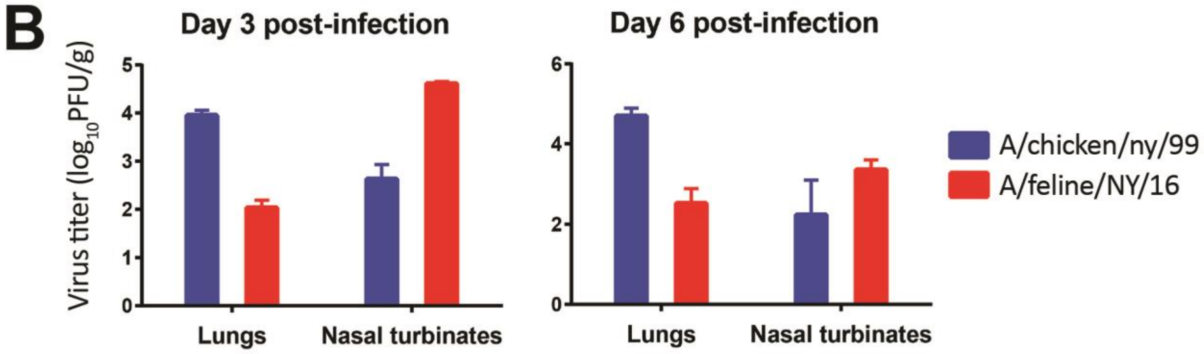

Technical Appendix Figure 11. Virus titers in the organs of infected mice. Six mice per group were infected intranasally with 10^5 PFU of A/feline/NY/16 and A/chicken/NY/99 viruses. Three mice in each group were euthanized on days 3 and 6 postinfection, and organs including brains, lungs, nasal turbinates, kidneys, livers, and spleens were collected. Viruses were isolated only from the lungs and nasal turbinates of infected animals; therefore, the other organs tested are not shown in the figure.

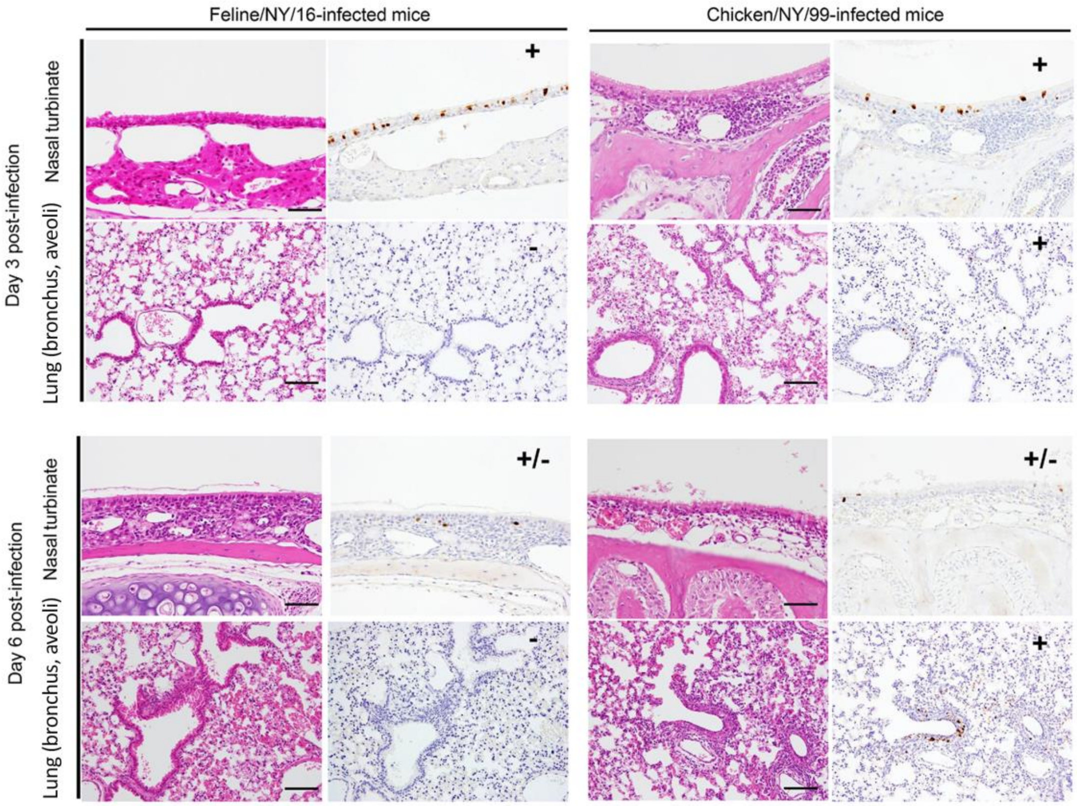

Technical Appendix Figure 12. Immunohistochemical findings in mice infected with A/feline/NY/16 or A/chicken/NY/99 virus. Shown are representative sections of nasal turbinates and lungs of mice infected with the indicated viruses on days 3 and 6 postinfection. Three mice per group were infected intranasally with 10^6 PFU of virus, and tissues were collected on days 3 and 6 post-infection. Influenza virus antigens were detected by a mouse monoclonal antibody for NP. For nasal turbinate sections: -, 0 NP-positive cells; +/–:,NP-positive cells detected in 1 focal region; +, NP-positive cells detected in >3 focal regions. For bronchus and alveolar sections: -, 0 NP-positive cells; +: >6 NP-positive cells. NP-positive cells were detected in focal, but not in diffuse bronchial and alveolar sections. For all analyses, the entire sections were evaluated. Left: H&E staining. Right: immunohistochemical staining for NP. Scale bars, 50 μm (nasal turbinates), 100 μm (lung).

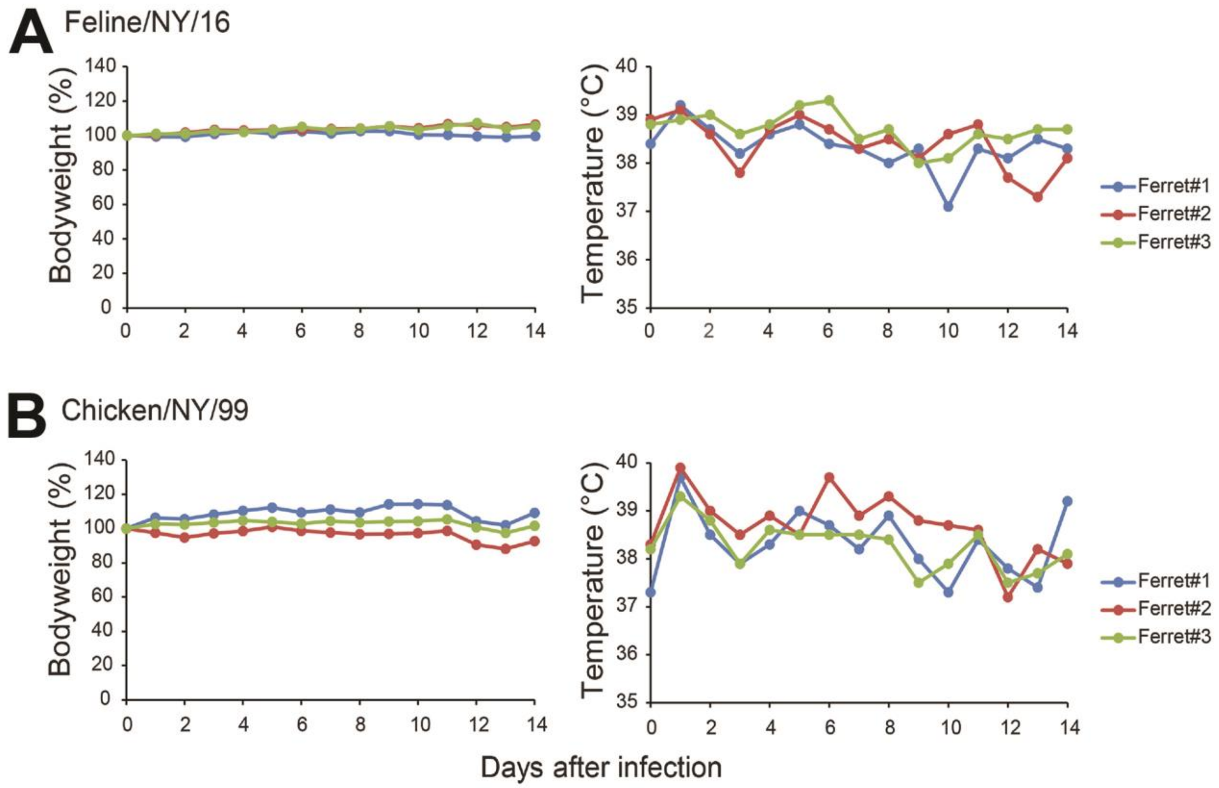

Technical Appendix Figure 13. Bodyweight and temperature changes in ferrets infected with 10^6 PFU of A/feline/NY/16 or A/chicken/NY/99 virus. Bodyweight and temperature were monitored daily for 14 days. A and B) Bodyweight and temperature changes for 3 ferrets per group infected with A/feline/NY/16 virus. C and D) Bodyweight and temperature changes for 3 ferrets per group infected with A/chicken/NY/99 virus.

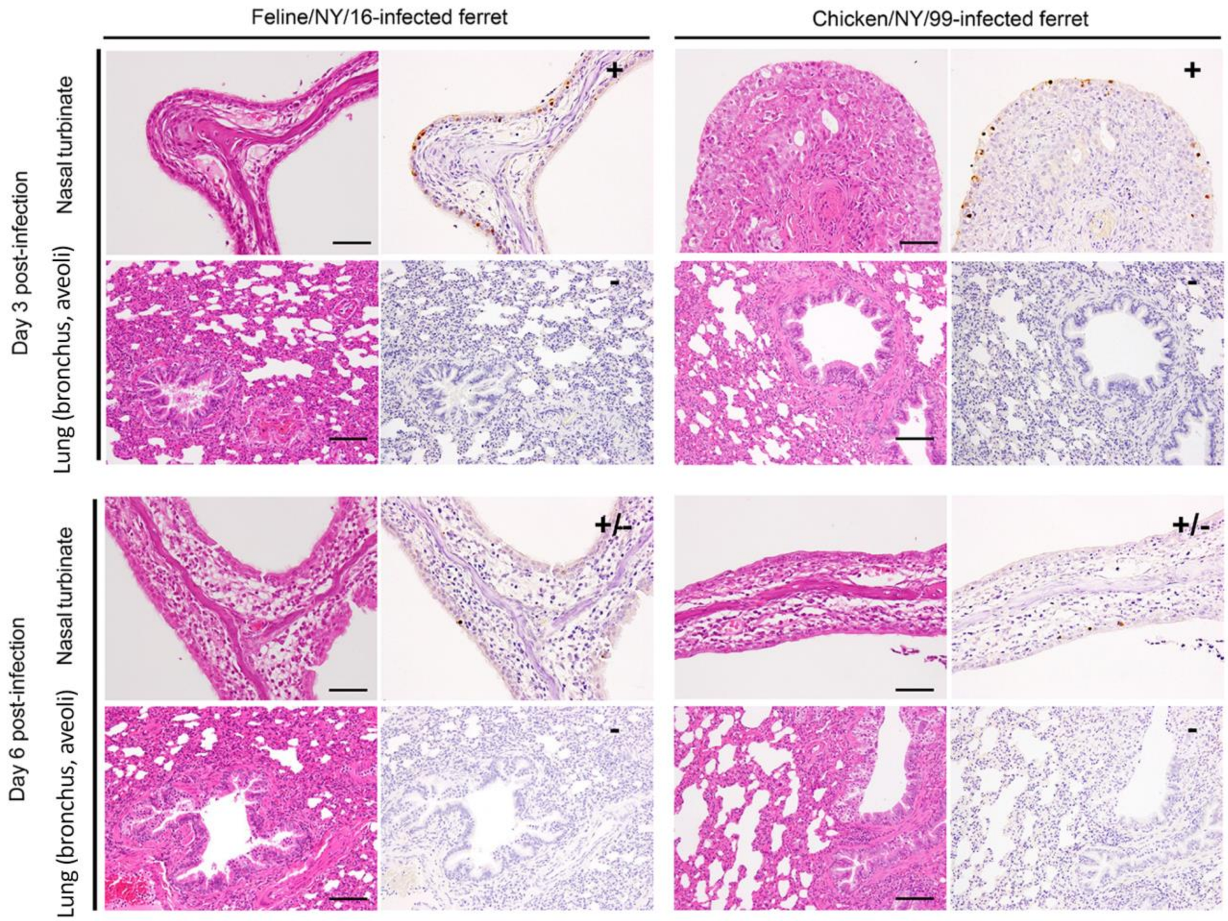

Technical Appendix Figure 14. Immunohistochemical findings in infected ferrets. Shown are representative sections of nasal turbinates and lungs of ferrets infected with the indicated viruses on days 3 and 6 postinfection. Three ferrets per group were infected intranasally with 10^6 PFU of virus, and tissues were collected on days 3 and 6 postinfection. Influenza virus nucleoprotein was detected by a rabbit polyclonal antibody to this protein. For nasal turbinate sections: -, 0 NP-positive cells; +/–:,NP-positive cells detected in 1 focal region; +, NP-positive cells detected in >3 focal regions. For bronchus and alveolar sections: -, 0 NP-positive cells. For all analyses, the entire sections were evaluated. Left: H&E staining. Right: immunohistochemical staining for NP. Scale bars, 50 μm (nasal turbinates), 100 μm (lung).
